# Chloride-Transporting OsHKT1;1 Splice Variants and Their Expression Profiles Under Salinity Stress in Rice

**DOI:** 10.3390/ijms27031178

**Published:** 2026-01-23

**Authors:** Shahin Imran, Shuntaro Ono, Rie Horie, Maki Katsuhara, Tomoaki Horie

**Affiliations:** 1 Institute of Plant Science and Resources, Okayama University, Kurashiki 710-0046, Okayama, Japan; ptj87a5q@s.okayama-u.ac.jp (S.I.);; 2 Department of Agronomy, Khulna Agricultural University, Khulna 9100, Khulna, Bangladesh; 3 Graduate School of Science and Engineering, Saitama University, Saitama 338-8570, Saitama, Japan; 4 Division of Applied Biology, Faculty of Textile Science and Technology, Shinshu University, Ueda 386-8567, Nagano, Japan

**Keywords:** HKT, heterologous expression systems, Na^+^ selectivity, Cl^−^ selectivity, rice

## Abstract

OsHKT1;1, a member of the high-affinity K^+^ transporter (HKT) family, plays a key role in Na^+^ homeostasis and salinity tolerance in rice. In our previous study, multiple potential *OsHKT1;1* splicing variants were identified, as well as the full-length (FL) *OsHKT1;1* transcript from the salt-tolerant rice Pokkali. However, most previous studies focused solely on the full-length protein, leaving the transport functions of splice variants largely unexamined. In this study, we focused on the splice variant OsHKT1;1-V2 and compared its function and gene expression with those of OsHKT1;1-FL. Two-electrode voltage clamp experiments using *Xenopus laevis* oocytes revealed that the 1st start codon of OsHKT1;1-V2 is functional to exhibit bidirectional currents in bath solutions containing NaCl. Unlike the Na^+^-selective feature of OsHKT1;1-FL, OsHKT1;1-V2 primarily mediated Cl^−^ transport with weak Na^+^ selectivity, which was supported by the higher Cl^−^ accumulation in OsHKT1;1-V2–expressing oocytes. Subcellular localization analyses using oocytes and *Arabidopsis* mesophyll cells indicated plasma membrane localization of OsHKT1;1-V2, similar to OsHKT1;1-FL. Functional assays using a yeast mutant further indicated that OsHKT1;1-FL, but not OsHKT1;1-V2, mediates Na^+^ uptake. The same *OsHKT1;1* variants were identified in the *japonica* cultivar Nipponbare, and OsHKT1;1-V2 of the cultivar showed Cl^−^ transport properties similar to the one from Pokkali. Quantitative PCR analyses revealed higher abundance of *OsHKT1;1-FL* transcripts in Nipponbare than in Pokkali with markedly lower *OsHKT1;1-V2* levels in Pokkali under salt stress. This study provides a new insight into HKT-mediated ion homeostasis under salinity stress.

## 1. Introduction

High concentrations of soluble or exchangeable salts (in most cases, the cation is Na^+^) inhibit plant growth under salinity stress, which is a globally alarming issue that significantly lowers the yield and development of major crops. Rice (*Oryza sativa*) is the most susceptible to salt stress among cereal crops [1,2]. When toxic Na ions are adequately excluded, redistributed, and compartmentalized in the plant body, the plants are likely to exhibit tolerance to salinity stress.

High-affinity K^+^ transporters (HKTs) are known to be involved in the transport of Na^+^ and K^+^ in plants, and some of them are crucial for maintaining Na^+^/K^+^ balance under salinity stress [3,4]. Therefore, HKTs have been widely examined since the first report of the *TaHKT2;1* gene in bread wheat (*Triticum aestivum*) [5]. HKTs are classified into two subfamilies, and transporters belonging to subfamily 1 (HKT1s) have generally been shown to be selective for Na^+^ [6,7,8,9]. The ability of plants to withstand salt has been demonstrated for both HKT1s and HKT2s.

In monocotyledon rice, the *japonica* cultivar was reported to maintain seven functional *HKT* genes, among which four genes (*OsHKT1;1*, *OsHKT1;3*, *OsHKT1;4*, and *OsHKT1;5*) were grouped in subfamily 1 [10,11]. Heterologous expression studies of OsHKT1;1 from rice using *Xenopus laevis* (*X. laevis*) oocytes showed Na^+^-selective transport in an inward-rectifying manner [8,12]. HKT1;1 transporters identified in various plant species exhibit robust Na^+^ selectivity when expressed in *Xenopus laevis* oocytes: AtHKT1;1 from *Arabidopsis* [7], HvHKT1;1 from barley [13], CmHKT1;1 from pumpkin [14], SvHKT1;1 from halophytic turf grass (*Sporobolus virginicus*) [15], VviHKT1;1 from grapevine [16], and VcHKT1;1 from blueberry [17]. Some HKT1 transporters interact with other ion homeostasis systems, such as the SOS pathway and K^+^ transporters [18,19], forming a coordinated regulatory network that maintains ionic balance and cellular stability under salinity stress [20,21]. OsHKT1;1 has been shown to be involved in limiting Na^+^ accumulation in leaves and contributing to salt tolerance based on the analysis of an insertional mutation in the *OsHKT1;1* gene [22]. Recent studies have further demonstrated that natural allelic variations in *OsHKT1;1* contribute significantly to genotypic differences in Na^+^ distribution within rice. Genome-wide association analysis using a global diversity panel identified *OsHKT1;1* as the gene underlying a major QTL (RNC4) that regulates root Na^+^ content, with indica accessions generally carrying alleles that promote higher root Na^+^ accumulation than japonica accessions [23]. Functional assays revealed that three indica-predominant non-synonymous substitutions increased the inward Na^+^ transport activity of OsHKT1;1, and introduction of the *indica* allele into a *japonica* background reproduced the high-root-Na^+^ phenotype. These results support earlier findings that *OsHKT1;1* limits Na^+^ accumulation in shoots and improves salt tolerance [22]. Furthermore, a combination of screening of salt-tolerant rice mutant lines and the MutMap method identified a key transcription factor gene that encodes a B-type response regulator OsRR22, suggesting the involvement of *OsHKT1;1* in rice salt tolerance [24].

In our previous study, an interesting phenomenon was found in the *OsHKT1;1* gene in a salt-tolerant landrace, Pokkali, in which potential alternative splicing variants encoding transporters that show distinct ion selectivity were identified in addition to the full-length *OsHKT1;1* transcript [8]. Alternative splicing (AS) is a post-transcriptional process that fine-tunes gene function. In rice, AS is broadly reprogrammed by salinity and other abiotic stresses, generating isoforms that adjust protein abundance and activity as conditions change [25]. This regulatory flexibility is evident in genes such as *OsDREB2B*, *OsHSFA2d*, and *OsMYB7*, where stress-dependent switching to functional splice variants enhances stress tolerance [25,26,27,28]. Similar patterns in other species reinforce the central role of AS in stress adaptation. In foxtail millet, for example, salt stress induces two *SiCYP19* splice variants, and *SiCYP19-b* more effectively improves tolerance by increasing proline and reducing ROS [29]. In *Populus euphratica*, AS of *PeuHKT1;3* generates the isoform *PeuHKT1;3a*, which alters pore-loop structure, enhances K^+^ transport, and improves K^+^/Na^+^ homeostasis [30]. In *Arabidopsis*, *At-SRAS1* produces two splice variants with opposite functions: *At-SRAS1.1* (full-length, active E3 ligase) enhances salt tolerance, while *At-SRAS1.2* (truncated) reduces it [31]. In the halophyte *Thellungiella salsuginea*, salt stress induces multiple splice variants of *TsHKT1;2*, among which the functional isoforms (TsHKT1;2a/2b) interact with other HKT1 proteins to reduce Na^+^ uptake and enhance salt tolerance [32]. In rice, *OsHKT1;4* also shows tissue-dependent splice variation that affects Na^+^ retrieval and supports ionic balance under salinity [33]. Despite these findings, AS regulation of HKTs in rice remains largely uncharacterized, and only a few other studies have reported splicing variants of HKTs [9,34].

Although alternative splicing of *OsHKT1;1* has been reported, the functional analyses of these variants, particularly whether they exhibit different transport characteristics from the full-length protein, remain unclear. Therefore, in the present study, we focused on one of the eight variants of the *OsHKT1;1* gene from Pokkali, *OsHKT1;1-V2*, to characterize its expression profile and transport properties. We present the results of heterologous expression analyses, which exhibit Cl^−^ transport features with far less selectivity for Na^+^ of OsHKT1;1-V2 than those of full-length OsHKT1;1. Furthermore, we showed evidence that identical potential splicing variants exist in a salt-sensitive *japonica* cultivar, Nipponbare, and that OsHKT1;1-V2 from Nipponbare also exhibits similar Cl^−^ transport properties, although the expression levels of the full-length and OsHKT1;1-V2 variant seemed to be differentially regulated between the two varieties. We provide new insights into the mechanisms of ion homeostasis in rice, mediated by the potential alternative splicing of an important salt tolerance gene, *OsHKT1;1*, and discuss questions to be elucidated for pursuing the physiological impacts.

## 2. Results

### 2.1. Identification of a Functional Frame in the OsHKT1;1-V2 Variant

**Figure 1:**
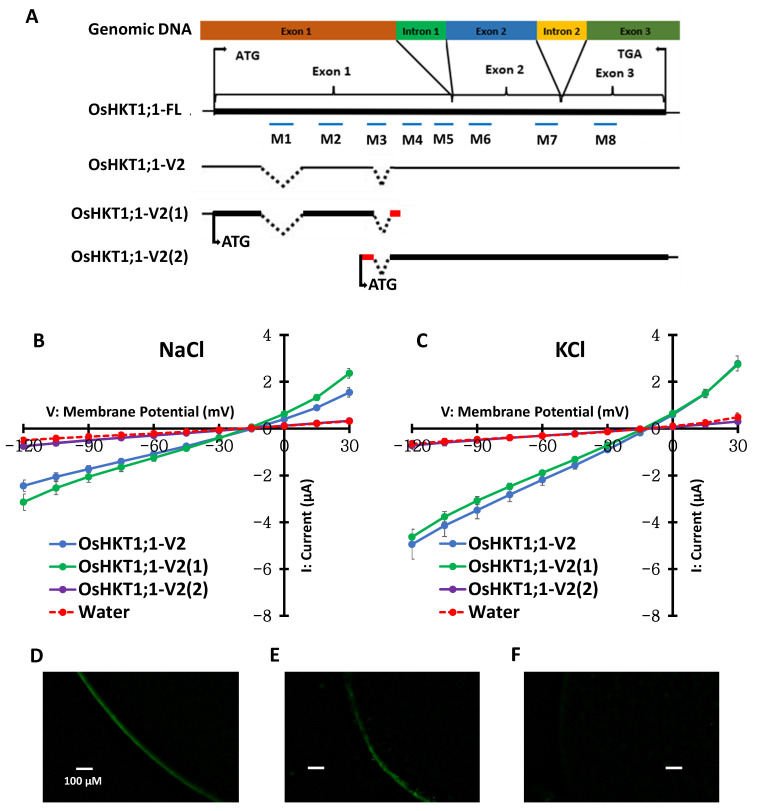
The first methionine functions as a start codon in the *OsHKT1;1-V2* variant. (**A**) Schematic representation of *OsHKT1;1-V2* mRNA, presumably produced by alternative splicing, and its artificially truncated fragments, *OsHKT1;1-V2(1)* and *OsHKT1;1-V2(2)*, each of which contains only one first methionine that could function as a start codon. Boxes indicate amino acid regions that are the same as those in OsHKT1;1-FL (black) or different from those in *OsHKT1;1-FL* (red) because of the frameshift. Dotted lines indicate missing nucleotide regions (gaps compared to the *OsHKT1;1-FL* sequence) due to alternative splicing events. The predicted transmembrane domains (M1–M8) were annotated based on previously curated information in the UniProt database. (**B**,**C**) Transport properties of OsHKT1;1-V2 and its artificial variants, characterized by two-electrode voltage clamp experiments using *X. laevis* oocytes immersed in external solutions containing 96 mM NaCl or 96 mM KCl. Data represent means ± SE, *n* = 15–18, from three independent experiments performed using different batches of oocytes. (**D**–**F**) Detection of fluorescence derived from the FLAG-tag in oocytes expressing FLAG-OsHKT1;1-FL (**D**) or FLAG-OsHKT1;1-V2 (**E**) or in oocytes injected with water (**F**). All external solutions contained 1.8 mM CaCl_2_, 1.8 mM MgCl_2_, 1.8 mM mannitol, and 10 mM HEPES (pH 7.5 with Tris) as background elements. Oocytes were isolated and injected with 50 ng cRNAs/50 nL or 50 nL of nuclease-free water as a negative control, and then incubated for approximately 4 h at 18 °C.

In our previous study, two start codons were identified in the *OsHKT1;1-V2* variant (Figure 1A) [8]. We artificially truncated and produced two fragments of *OsHKT1;1-V2* named *OsHKT1;1-V2(1)* and *OsHKT1;1-V2(2)*. *OsHKT1;1-V2(1)* is composed of 519 nucleotides, encoding 172 putative amino acid residues. In contrast, *OsHKT1;1-V2(2)* is composed of 957 nucleotides encoding 318 putative amino acid residues (Figure 1A, Supplementary Figure S1). *X. laevis* oocytes were injected with cRNAs of *OsHKT1;1-V2*, *OsHKT1;1-V2(1)*, and *OsHKT1;1-V2(2)*, and TEVC experiments were performed to identify which fragment induces bidirectional ionic currents like OsHKT1;1-V2 [8]. OsHKT1;1-V2(1) but not OsHKT1;1-V2(2) showed ionic currents in both NaCl and KCl bath solutions as OsHKT1;1-V2 (Figure 1B,C). These results suggest that the start codon of OsHKT1;1-V2 is the 1st methionine. Furthermore, histochemical analysis indicated that FLAG tag-fused OsHKT1;1-FL and OsHKT1;1-V2 proteins were localized in the plasma membrane (PM) of oocytes (Figure 1D,E). In contrast, control oocytes injected with water showed no robust green fluorescence in the PM (Figure 1F).

### 2.2. Subcellular Localization of GFP-Fused OsHKT1;1-FL and OsHKT1;1-V2 in Plant Cells

**Figure 2:**
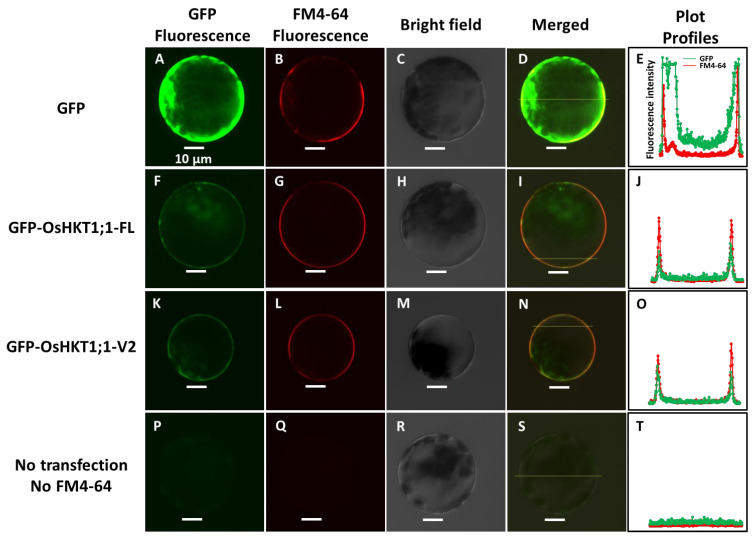
Subcellular localization of GFP-fused OsHKT1;1 transporters in *Arabidopsis* protoplasts. GFP (**A**–**D**), GFP-OsHKT1;1-FL (**F**–**I**), or GFP-OsHKT1;1-V2 (**K**–**N**) was transiently overexpressed in *Arabidopsis* leaf protoplasts. The cells were incubated with FM4-64, which was used as a PM marker. Protoplasts that were not transfected or treated with FM4-64 (**P**–**S**) were used as negative controls. Plot-profile fluorescence intensity analysis of protoplasts expressing different constructs (red and green colors represent fluorescence from FM4-64 and GFP, respectively): GFP (**E**), GFP-OsHKT1;1-FL (**J**), GFP-OsHKT1;1-V2 (**O**), and untransfected and no FM4-64-treated protoplasts (**T**).

To study the subcellular localization in plant cells, green fluorescence protein (GFP) was fused with OsHKT1;1-FL and OsHKT1;1-V2 at the N-terminus. DNA constructs to express either GFP, GFP-OsHKT1;1-FL, or GFP-OsHKT1;1-V2 were introduced into *Arabidopsis* leaf protoplasts [35]. Cells expressing GFP showed strong fluorescence in the cytosol, which did not overlap with the plasma membrane marker FM4-64 (Figure 2A–D). GFP fluorescence from cells expressing GFP-OsHKT1;1-FL was observed at the cell periphery, overlapping with FM4-64 fluorescence (Figure 2F–I). A similar overlap of GFP and FM4-64 fluorescence was observed in cells expressing GFP-OsHKT1;1-V2 (Figure 2K–N). Plot-profile fluorescence intensity analysis of the merged fluorescence images supported the above-mentioned observation (Figure 2E,J,O). Together with the negative control experiments shown in Figure 2P–S, these results suggest the plasma membrane localization of OsHKT1;1-V2 as OsHKT1;1-FL.

### 2.3. Substrate Selectivity of OsHKT1;1-FL and OsHKT1;1-V2

**Figure 3:**
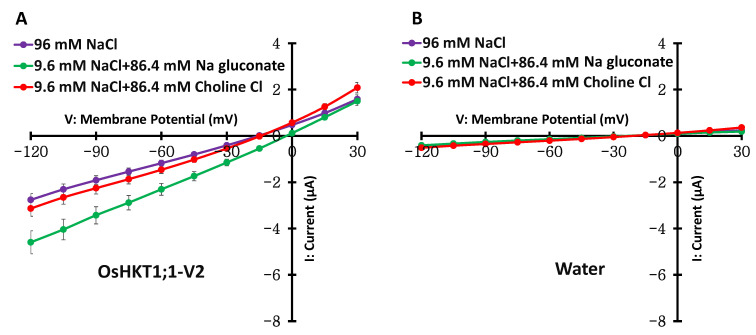
Current–voltage relationships of OsHKT1;1-V2 (**A**) expressed in *X. laevis* oocytes and the oocytes injected with water (**B**). The external solutions used were 96 mM NaCl, 9.6 mM NaCl + 86.4 mM Na gluconate, or 9.6 mM NaCl + 86.4 mM Choline Cl. All external solutions contain, as background elements, 1.8 mM CaCl_2_, 1.8 mM MgCl_2_, 1.8 mM mannitol, and 10 mM HEPES (pH 7.5 with Tris). Oocytes were injected with 50 ng cRNAs/50 nL and then incubated for approximately 4 h (OsHKT1;1-V2) or 18 h (water-injected oocytes) at 18 °C before TEVC measurements (note that incubation of OsHKT1;1-V2-expressing oocytes for more than 4 h made it difficult to use them for TEVC experiments due to apparent damage). Data are presented as means ± SE, *n* = 10–15, from two independent experiments performed with different batches of oocytes.

Current–voltage relationships were obtained in the presence of 96 mM NaCl (96 mM Na^+^ and 96 mM Cl^−^), 9.6 mM NaCl + 84.6 mM Na gluconate (total 96 mM Na^+^ and 9.6 mM Cl^−^), and 9.6 mM NaCl + 84.6 mM Choline Cl (9.6 mM Na^+^ and total 96 mM Cl^−^) by conducting electrophysiological TEVC experiments using *X. laevis* oocytes. As described previously [12], a decrease in Na^+^ concentration from 96 mM to 9.6 mM largely reduced the inward currents in OsHKT1;1-FL and showed no tendency to produce robust outward currents (Supplementary Figure S2). In contrast, the same change in solution did not affect OsHKT1;1-V2-mediated currents (Figure 3A). Next, we changed the Cl^−^ concentration of the bath solution while keeping the Na^+^ concentration at 96 mM during TEVC measurements. The 10 times reduction in the Cl^−^ concentration led to an increase in OsHKT1;1-V2-mediated currents with a positive shift in the reversal potential of 13 mV (Figure 3A, purple and green lines). Note that similar large currents were never observed in water-injected oocytes under any bath condition (Figure 3B).

Next, oocytes expressing OsHKT1;1-V2 were analyzed in 96 mM NaCl (96 mM Na^+^ and 96 mM Cl^−^), 96 mM Na gluconate (total 96 mM Na^+^ and 0 mM Cl^−^), and 96 mM Choline Cl (0 mM Na^+^ and total 96 mM Cl^−^) solutions with voltage steps from +90 to −120 mV in 15 mV decrements to examine outward currents. OsHKT1;1-V2 expressed in oocytes exhibited large outward currents with no reversal potential shift when the solution was changed from 96 mM NaCl to 96 mM Choline Cl, by which Na^+^ concentration was decreased from 96 mM to 0 mM (Supplementary Figure S3A, purple and red lines). In contrast, reversal potential shifts occurred according to a change in Cl^−^ concentration from 96 mM to 0 mM in OsHKT1;1-V2 (Supplementary Figure S3A, purple and red lines vs. green line). Note that endogenous outward currents were also observed in water-injected oocytes, but they were never the same extent as OsHKT1;1-V2-expressing oocytes in every bath condition (Supplementary Figure S3B). These results suggest that the currents induced by OsHKT1;1-V2 largely depend on Cl^−^.

### 2.4. Substrate Concentration Dependency of OsHKT1;1-FL and OsHKT1;1-V2

**Figure 4:**
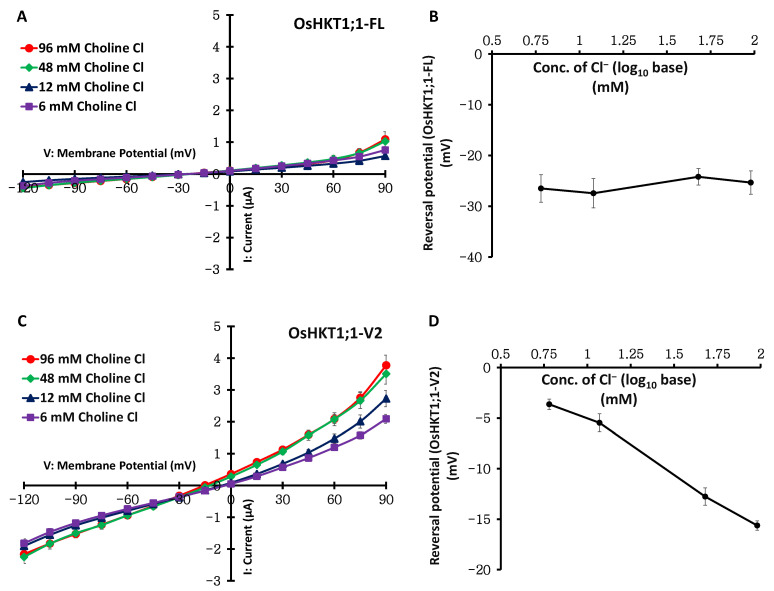
Chloride concentration dependency of OsHKT1;1-FL and OsHKT1;1-V2 expressed in *X. laevis* oocytes. (**A**,**C**) Current–voltage relationships of oocytes expressing OsHKT1;1-FL and OsHKT1;1-V2. (**B**,**D**) Log10 base reversal potential analysis of OsHKT1;1-FL and OsHKT1;1-V2, respectively. A series of choline chloride solutions with concentrations ranging from 96 mM to 6 mM was used as the external bath solution. The basic components of the solutions were 1.8 mM CaCl_2_, 1.8 mM MgCl_2_, 1.8 mM mannitol, and 10 mM HEPES, and the pH was adjusted to 7.5 using Tris. The 48, 12, and 6 mM choline chloride solutions contained 38, 63, and 68 mM Mg gluconate, respectively. Oocytes were isolated and injected with 2.5 ng cRNAs/50 nL and then incubated for approximately 18 h for OsHKT1;1-FL or 4 h for OsHKT1;1-V2 at 18 °C. To obtain current–voltage relationships, voltage steps (2 s) were applied from +90 to −120 mV in 15 mV decrements. Data are presented as mean ± SE, *n* = 16–18, from two independent experiments using different batches of oocytes.

**Figure 5:**
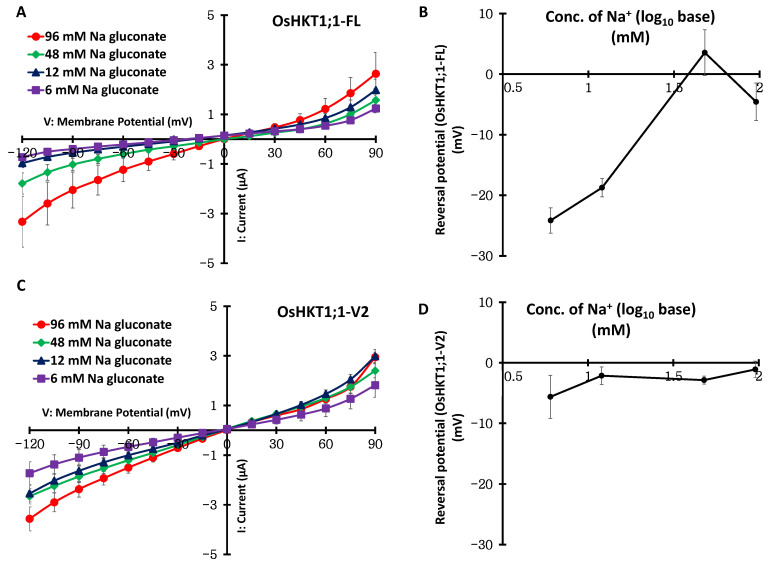
Sodium concentration dependency of OsHKT1;1-FL and OsHKT1;1-V2 expressed in *X. laevis* oocytes. (**A**,**C**) Current–voltage relationships from oocytes expressing OsHKT1;1-FL and OsHKT1;1-V2, respectively. (**B**,**D**) Log10 base reversal potential analysis of OsHKT1;1-FL and OsHKT1;1-V2, respectively. A series of sodium gluconate solutions with concentrations ranging from 96 mM to 6 mM was used as the external bath solution. The basic components of the solutions contained 1.8 mM Ca gluconate, 1.8 mM Mg gluconate, 1.8 mM mannitol, and 10 mM HEPES, and the pH was adjusted to 7.5 using Tris. The 48, 12, and 6 mM sodium gluconate solutions contained 38, 63, and 68 mM Mg gluconate, respectively. Oocytes were isolated and injected with 2.5 ng cRNAs/50 nL and then incubated for about 18 h for OsHKT1;1-FL or 4 h for OsHKT1;1-V2 at 18 °C. To obtain current–voltage relationships, voltage steps (2 s) were applied from +90 to −120 mV in 15 mV decrements. Data are presented as mean ± SE, *n* = 15–16, from two independent experiments using different batches of oocytes.

Further TEVC measurements were performed using oocytes expressing OsHKT1;1-FL or OsHKT1;1-V2 in a series of bath solutions that contained Cl^−^ as the sole permeable monovalent anion (96, 48, 12, and 6 mM as choline chloride) or Na^+^ as the sole permeable monovalent cation (96, 48, 12, and 6 mM as Na gluconate). OsHKT1;1-FL-expressing oocytes showed currents comparable to those of water-injected control oocytes, with no reversal potential shift in a series of changes in Cl^−^ concentration (Figure 4A,B, Supplementary Figure S4B). However, OsHKT1;1-FL showed large and various currents with reversal potential shifts in a series of changes in the Na^+^ concentration (Figure 5A,B). These data suggest that OsHKT1;1-FL mediates the transport of Na^+^. In contrast, oocytes expressing OsHKT1;1-V2 exhibited robust inward and outward currents with reversal potential shifts in a series of changes in the Cl^−^ concentration (Figure 4C,D). In contrast, OsHKT1;1-V2-expressing oocytes showed larger currents than water-injected control oocytes in accordance with a series change in the Na^+^ concentration; however, the reversal potential did not alter as in oocytes expressing OsHKT1;1-FL (Figure 5B–D, Supplementary Figure S4A). These data suggest that, unlike OsHKT1;1-FL, OsHKT1;1-V2 is selective for Cl^−^ and mediates its transport.

### 2.5. Cl^−^ Contents in Oocytes

**Figure 6:**
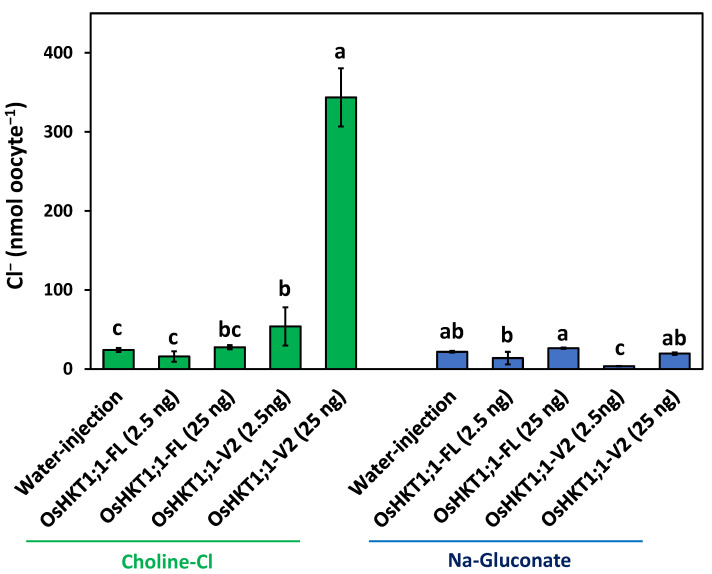
Cl^−^ contents of oocytes injected with *OsHKT1;1-FL* cRNA, *OsHKT1;1-V2* cRNA, or water. Oocytes were treated with 96 mM Na-gluconate or 96 mM choline-Cl solution for 6 h at room temperature. See Section 4 for details. Data are shown as mean  ±  SD. 4 to 8 batches of oocytes were used to measure Cl^−^ contents (note that each batch included 8–10 oocytes): *n* = 8 batches for OsHKT1;1-FL (2.5 ng), 8 for OsHKT1;1-FL (25 ng), 8 for OsHKT1;1-V2 (2.5 ng), 8 for OsHKT1;1-V2 (25 ng), and 8 for water injection in a choline Cl treatment; and *n* = 8 batches for OsHKT1;1-FL (2.5 ng), 4 for OsHKT1;1-FL (25 ng), 4 for OsHKT1;1-V2 (2.5 ng), 4 for OsHKT1;1-V2 (25 ng), and 8 for water injection in a Na-gluconate treatment. A one-way ANOVA with Tukey post hoc test was used for the Choline-Cl and Na-Gluconate group separately; different lowercase letters indicate statistically significant differences (*p  <*  0.05).

Cl^−^ accumulation was investigated using oocytes injected with water, *OsHKT1;1-FL* cRNA, or *OsHKT1;1-V2* cRNA, which were incubated in 96 mM choline chloride-containing medium (Figure 6). The total Cl^−^ content showed that OsHKT1;1-V2-expressing oocytes (2.5 ng cRNA) accumulated significantly more Cl^−^ than OsHKT1;1-FL-expressing (2.5 ng cRNA) and water-injected oocytes (Figure 6). Furthermore, a 10-fold increase in the injection volume of the *OsHKT1;1-V2* cRNA (25 ng) resulted in the accumulation of approximately 6 times more Cl^−^ (Figure 6). To verify that the observed Cl^−^ increase was specifically due to Cl^−^ influx and not caused by nonspecific anion accumulation, the same experiment was performed in Na-gluconate medium without Cl^−^. Under these Cl^−^-free conditions, all oocytes, including those expressing OsHKT1;1-V2, showed low Cl^−^ levels (Figure 6). These results support that OsHKT1;1-V2 mediates Cl^−^ transport in the PM of oocytes, as suggested by the TEVC experiments shown in Figure 3 and Figure 4.

### 2.6. Functional Characterization of OsHKT1;1 Transporters in Yeast

**Figure 7:**
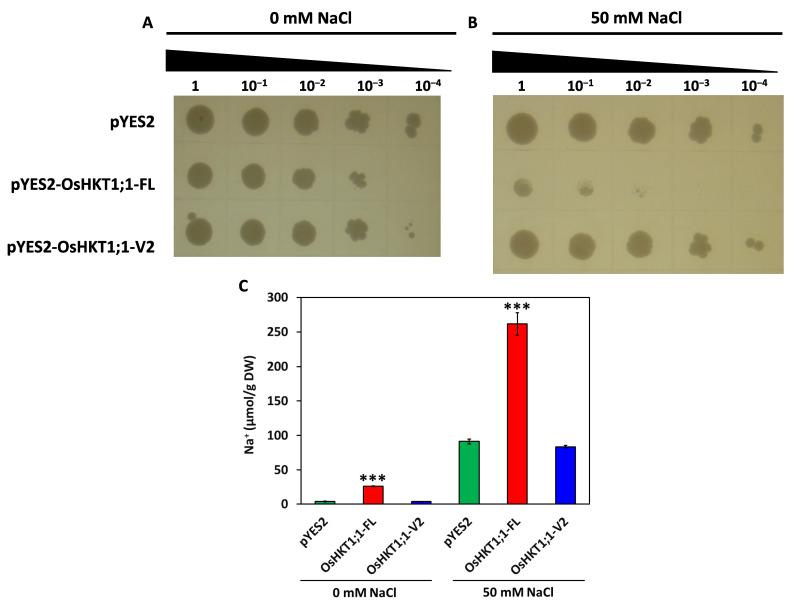
Functional characterizations of *OsHKT1;1-FL* and *OsHKT1;1-V2* in the G19 yeast strain. Growth of G19 cells transformed with empty vector (pYES2), pYES2-*OsHKT1;1-FL*, and pYES2-*OsHKT1;1-V2*. Each G19 transformant was incubated at 30 °C for 4 days on -Ura medium plates [0.67% (*w*/*v*) yeast nitrogen base without amino acids, 0.077% (*w*/*v*) drop out mix-Ura, 2% (*w*/*v*) galactose, 1% (*w*/*v*) raffinose, and 2% agar] containing 0 mM NaCl (**A**) or 50 mM NaCl (**B**). Numbers (1, 10^−1^, 10^−2^, 10^−3^, 10^−4^) on the top of each panel indicate the serial dilutions (OD_600_) of yeast cells spotted on the medium. (**C**) Na^+^ content of G19 transformants that were incubated in liquid synthetic complete (SC) medium supplemented with or without 50 mM NaCl for 18 (control) and 21 (NaCl-treated) h at 30 °C (*n* = 5, ± SD). Asterisks denote significant differences from control (pYES2 empty vector) (Student’s *t*-test: *** *p* < 0.001).

*OsHKT1;1-FL* and *OsHKT1;1-V2* were introduced into the Na^+^-sensitive mutant yeast strain G19 to further examine the Na^+^ transport capacity of *OsHKT1;1-FL* and *OsHKT1;1-V2*. No remarkable difference was found in the growth among *OsHKT1;1-FL*, *OsHKT1;1-V2*, and empty vector-harboring G19 cells under the control condition (Figure 7, left panel). However, the presence of 50 mM NaCl caused severe growth defects in OsHKT1;1-FL-expressing cells. In contrast, cells expressing *OsHKT1;1-V2* grew similarly to the vector-harboring control cells (Figure 7, right panel). These data indicate that *OsHKT1;1-FL*, but not *OsHKT1;1-V2*, possibly accelerated Na^+^ uptake in yeast cells, leading to toxicity and growth reduction in the cells under NaCl-added conditions. Thus Na^+^ contents of G19 transformants were determined under no or 50 mM NaCl conditions (Figure 7C). After 18 and 21 h incubation, for control and NaCl-added conditions, respectively, in the presence of galactose, G19 cells expressing OsHKT1;1-FL were found to accumulate significantly higher levels of Na^+^ than the others in both conditions (Figure 7C). Whereas Na^+^ contents of vector control and OsHKT1;1-V2 lines were at similar levels (Figure 7C). These results were consistent with the growth phenotype (50 mM NaCl; Figure 7B).

### 2.7. Transport Properties of OsHKT1;1-FL and Variants from Nipponbare

**Figure 8:**
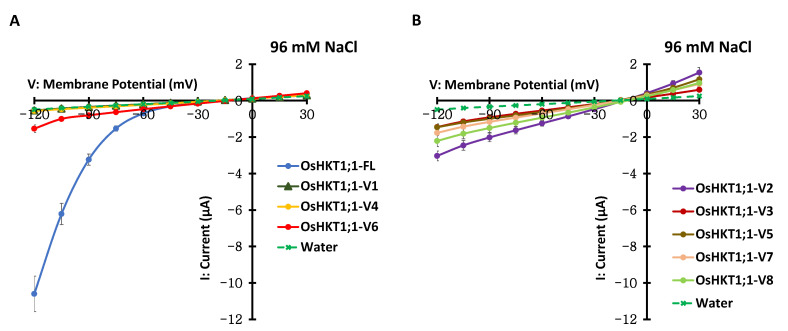
TEVC recordings of oocytes expressing OsHKT1;1-FL and OsHKT1;1 variants derived from cv. Nipponbare. (**A**) Current–voltage relationships from oocytes expressing OsHKT1;1-FL, -V1, -V4, and-V6 and oocytes injected with water. (**B**) Current–voltage relationships from oocytes expressing OsHKT1;1-V2, -V3, -V5, -V7, and-V8 and oocytes injected with water. The external bath solution contained 96 mM NaCl, 1.8 mM CaCl_2_, 1.8 mM MgCl_2_, 1.8 mM mannitol, and 10 mM HEPES (pH 7.5, with Tris). Data are presented as mean ± SE, *n* = 10–18, from two independent experiments using different batches of oocytes.

In a previous study [8] and in Figure 1, Figure 2, Figure 3, Figure 4, Figure 5, Figure 6 and Figure 7 in the present study, possible *OsHKT1;1* splicing variants, in addition to the full-length cDNA from salt-tolerant *indica* rice, Pokkali, were investigated. Therefore, we attempted to determine whether these *OsHKT1;1* splicing variants could also be found in the salt-sensitive *japonica* rice, Nipponbare, and whether they functioned in a manner similar to those from Pokkali. *OsHKT1;1* cDNAs were isolated from Nipponbare, and their transport properties were examined by TEVC experiments. Eight possible *OsHKT1;1* variants were detected in addition to the full-length cDNA from Nipponbare as Pokkali [8]. Comparative analysis revealed amino acid substitutions in OsHKT1;1 between the two genotypes (Supplementary Figure S5A–I). However, oocytes expressing Nipponbare (Ni) OsHKT1;1-FL and OsHKT1;1-V6 exhibited inward-rectifying currents in the presence of 96 mM NaCl, with much smaller currents of OsHKT1;1-V6 than OsHKT1;1-FL (Figure 8A). In contrast to Ni-OsHKT1;1-V1 and -V4, whose currents were similar to those from water-injected control oocytes (Figure 8A), Ni-OsHKT1;1-V2, -V3, -V5, -V7, and-V8 mediated ionic currents in a bidirectional manner, depending on the membrane voltage (Figure 8B). There was no remarkable difference in the pattern of elicited currents mediated by these variants between Pokkali and Nipponbare.

Next, oocytes expressing Ni-OsHKT1;1-FL and Ni-OsHKT1;1-V2 were examined in Cl^−^ solutions (96, 48, 12, and 6 mM as choline chloride) (Supplementary Figure S6). Current–voltage relationships obtained from Ni-OsHKT1;1-FL expressed in oocytes showed small or no currents and no reversal potential shift with changes in Cl^−^ concentrations in the bath solution (Supplementary Figure S6A,B). In contrast, oocytes injected with *OsHKT1;1-V2* cRNA exhibited both inward and outward currents with apparent reversal potential shifts according to the Cl^−^ concentration (Supplementary Figure S6C,D). These data suggest that Ni-OsHKT1;1-V2, but not Ni-OsHKT1;1-FL, mediates Cl^−^ transport, as observed in those transporters from Pokkali.

### 2.8. Expression Analyses of OsHKT1;1-FL and OsHKT1;1-V2 in Shoots and Roots

**Figure 9:**
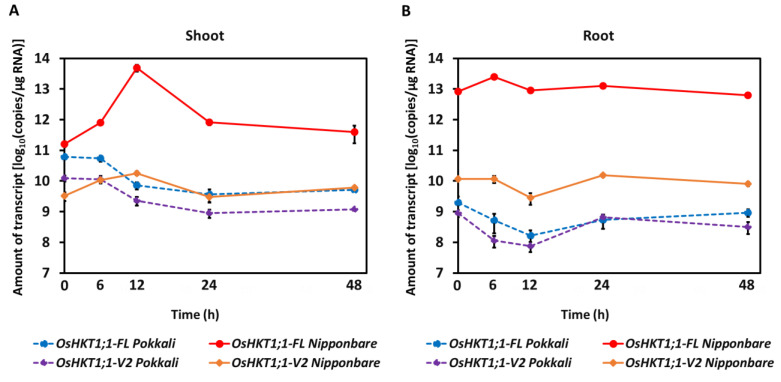
Absolute quantification of *OsHKT1;1-FL* and *OsHKT1;1-V2* transcript levels in Pokkali and Nipponbare plants with or without salt stress. The number of transcripts is displayed in log10 base in shoots (**A**) and roots (**B**). Fourteen-day-old seedlings of both varieties were treated with or without 100 mM NaCl (6–48 h) before total RNA extraction. Data are presented as the mean ± SE (*n* = 6).

We next investigated the transcript levels of *OsHKT1;1-FL* and *OsHKT1;1-V2* in Pokkali and Nipponbare by conducting qPCR-based absolute quantification. Without stress, Nipponbare plants accumulated approximately five times more *OsHKT1;1-FL* transcripts than Pokkali in shoots (Figure 9A). However, in the case of *OsHKT1;1-V2*, the transcript level in shoots was approximately six times higher in Pokkali than in Nipponbare (Figure 9A at time 0; note that the scale for transcript amounts is log-10-based). Interestingly, salt stress (100 mM NaCl) temporally increased both *OsHKT1;1-FL* and *OsHKT1;1-V2* transcripts in Nipponbare, peaking at 12 h, whereas those of Pokkali decreased (Figure 9A). In roots, the *OsHKT1;1-FL* transcript level was much lower in Pokkali than in Nipponbare, and so was the level of the *OsHKT1;1-V2* transcript under control conditions (Figure 9B). The OsHKT1;1-FL transcript level was temporally increased, peaking at 6 h after salt stress in Nipponbare, but this observation was not applied to Pokkali (Figure 9B). The levels of the other transcripts were maintained relatively constant during the periods investigated (Figure 9B). These results indicate a differential regulatory mechanism of *OsHKT1;1* gene expression between rice genotypes.

## 3. Discussion

HKT membrane proteins are known to be relevant to Na^+^ homeostasis in plants, including under salinity stress. Na^+^ exclusion protects photosynthetic tissues from salt-induced damage [6], and some of the Na^+^-selective class I HKT (HKT1) transporters play a key role in excluding Na^+^ from leaves, enhancing salinity tolerance [4,22,36,37,38,39]. In rice, OsHKT1;1 and OsHKT1;3 exhibit strong and weak inward rectification for Na^+^ transport, respectively [8,9,12]. Many other studies on the homologue of HKT1;1 from barley, pumpkin, grapevine, blueberry, and wild turf *Sporobolus virginicus* have also revealed Na^+^-selective transport as OsHKT1;1 [13,14,15,16,17]. Campbell et al. [23] showed that natural allelic variants of *OsHKT1;1* underlie the divergence in root Na^+^ content between japonica and indica rice cultivars. They reported three non-synonymous mutations associated with higher OsHKT1;1 activity and Na^+^ transport efficiency, explaining the genetic basis for the diversity in salt tolerance in rice. This study also provides strong genetic evidence that *OsHKT1;1* plays a central role in Na^+^ homeostasis and is an important target for improving salinity tolerance through molecular breeding. Additionally, *OsHKT1;1* interacts with the Salt Overly Sensitive (SOS) pathway and other transporters, implying a complex regulatory network for ion balance and salt stress response [11,18,20,40].

In our previous study, eight *OsHKT1;1* variants (V1-V8) that could be the product of alternative splicing were identified in addition to the full-length *OsHKT1;1-FL* cDNA in the salt-tolerant rice cultivar Pokkali [8]. In this study, we focused on the variant *OsHKT1;1-V2* as the transcript level was significantly higher than that of *OsHKT1;1-FL* in shoots of Pokkali, and *OsHKT1;1-V2* cRNA-injected oocytes exhibited larger inward and outward currents than other variants in both NaCl and KCl bath solutions [8]. Although two start codons are present in *OsHKT1;1-V2* mRNA, the present study demonstrated that the first start codon of *OsHKT1;1-V2* is functional (Figure 1A–C). The plasma membrane localization of FLAG-tagged OsHKT1;1-V2 in oocytes (Figure 1E) indicated that the ionic currents elicited by OsHKT1;1-V2 (Figure 1, Figure 3, Figure 4 and Figure 5) were not artifacts or leaks. In the present study, the co-localization of GFP-fused OsHKT1;1-FL or OsHKT1;1-V2 with the dye FM4-64 in the plasma membrane was further demonstrated in *Arabidopsis* protoplasts (Figure 2), supporting the above-mentioned notion.

OsHKT1;1-FL was confirmed to mediate Na^+^ transport, as reported previously [8] (Figure 7, Supplementary Figure S2). Interestingly, however, OsHKT1;1-V2 displayed distinct selectivity for Na^+^ and Cl^−^: the OsHKT1;1-V2 protein expressed in oocytes was responsive to the changes in the Cl^−^ concentration but not the Na^+^ concentration, suggesting a preference for Cl^−^ transport over Na^+^ transport (Figure 3, Supplementary Figure S3). OsHKT1;1-FL showed a concentration-dependent reversal potential shift only in Na^+^ bath solutions, whereas OsHKT1;1-V2 showed no concentration-dependent reversal potential shifts in Na^+^ solutions (Figure 5, Supplementary Figure S7). In contrast to the lack of reversal potential shifts in OsHKT1;1-V2-expressing oocytes upon Na^+^ concentration changes, the observed apparent reversal potential shifts upon Cl^−^ concentration changes again suggested that OsHKT1;1-V2 is more selective for Cl^−^ (Figure 4 and Figure 5). This electrophysiological suggestion was further supported by the measurements of Cl^−^ contents of oocytes expressing OsHKT1;1-V2, which accumulated significantly more Cl^−^ than the oocytes expressing OsHKT1;1-FL after incubation in the examination solution (Figure 6). In fact, oocytes expressing OsHKT1;1-V2 (2.5 ng cRNA) in the medium exhibited an average reversal potential of −17 mV (Supplementary Figure S3A), while the resting potential of the oocytes was approximately −9 mV, which was +8 mV higher than the reversal potential, indicating that Cl^−^ influx can be triggered under the tested conditions. The obtained results were consistent with those from the TEVC analysis (Figure 3, Figure 4 and Figure 5, Supplementary Figure S3). On the other hand, functional assays using the yeast G19 strain supported the notion that OsHKT1;1-FL, but not OsHKT1;1-V2, mediates Na^+^ uptake (Figure 7). In addition to the growth phenotype, quantification of Na^+^ levels in yeast cells provided evidence that OsHKT1;1-FL but not OsHKT1;1-V2 led to overaccumulation of Na^+^ even under a control condition where the existence of 1 mM or less Na^+^ is expected (Figure 7C). This result confirms that the growth inhibition observed in OsHKT1;1-FL-expressing G19 strain is attributable to enhanced Na^+^ uptake. More importantly, the lack of remarkable Na^+^ accumulation in OsHKT1;1-V2-expressing cells is fully consistent with the electrophysiological analyses in *Xenopus* oocytes (Figure 3 and Figure 5).

During salt stress, maintenance of Cl^−^ homeostasis is crucial because excess cytosolic Cl^−^ can be toxic [41,42]. Cl^−^ detoxification involves xylem loading, vacuolar sequestration, and efflux from roots [41,43]. Chloride channels (CLCs) are membrane proteins that mediate Cl^−^ and NO_3_^−^ transport across plant membranes, contributing to pH regulation and stress tolerance [44,45]. Each CLC monomer contains 16–18 transmembrane α-helices that form a self-contained anion pore and a cytosolic CBS domain that regulates gating through conformational and nucleotide-dependent mechanisms [45,46]. The conserved GxGxPE and GKxGPxxH motifs form part of the selectivity filter, where serine or proline residues determine Cl^−^ versus NO_3_^−^ specificity [47]. Overexpression of *GmCLC1*, *CsCLC-c*, and *ZmCLC-d* has been reported to enhance salt tolerance by promoting vacuolar Cl^−^ sequestration and reducing cytosolic accumulation [48,49,50]. The plasma membrane transporters NPF2.4 and SLAH1 are proposed to function in regulating Cl^−^ loading into the xylem [51,52], whereas tonoplast-localized CLCs mediate Cl^−^/H^+^ antiport to sequester Cl^−^ into vacuoles [41,42]. Additionally, *AtCCC1* functions as a Na^+^:K^+^:Cl^−^ cotransporter essential for maintaining ion balance and osmotic potential [53]. Loss-of-function mutants of *AtCCC1* and *OsCCC1* exhibit stunted growth and excessive Cl^−^ accumulation in shoots, indicating that CCC1 participates in Cl^−^ retrieval and homeostasis [53,54]. Moreover, some ALMT members (*AtALMT9* and *AtALMT12*) facilitate Cl^−^ transport [55,56].

In the present study, we demonstrated that the OsHKT1;1-V2 splice variant encodes a protein of 172 amino acids with a single predicted transmembrane helix (Supplementary Figure S1A). Despite lacking the canonical pore-forming domains of HKTs, TEVC assays revealed robust inward and outward currents, suggesting that OsHKT1;1-V2 may form a self-associated complex that is permeable to Cl^−^. Sequence inspection of OsHKT1;1-V2 revealed a central hydrophobic stretch (residues 96–118; “LWVLILLMLMGGEVFTSMLGLYF”) (Supplementary Figure S1A), likely forming the ion-conducting α-helix, surrounded by charged and polar residues that could stabilize Cl^−^ within the pore. Notably, OsHKT1;1-V2 contains an upstream GKxGPxx pattern (residues 61–67) that resembles part of the conserved GKxGPxxH selectivity motif present in plant CLC proteins (Supplementary Figure S1A). Although this pattern lacks the canonical histidine present in CLC selectivity filters, it may still contribute to local structural flexibility or pore shaping, providing a residual signature of the anion-interacting architecture. Interestingly, the overall minimal architecture of OsHKT1;1-V2 parallels the basic structural layout of inwardly rectifying K^+^ (Kir) channels, which are composed of two transmembrane helices (TM1 and TM2) connected by a pore-forming loop and assembled as a tetramer to form a functional channel [57]. Kir channels exhibit inward rectification through pore blockage by intracellular Mg^2+^ and polyamines, allowing greater ion influx than efflux [57]. By analogy, OsHKT1;1-V2 might represent a minimal anion-conducting module, in which its single transmembrane helix or multimeric assembly provides a rudimentary Cl^−^ pathway.

Although *Xenopus* oocytes have endogenous anion channels, the following facts minimize the possibility that these channels contributed to the OsHKT1;1-V2-associated currents. First, in contrast to the Cl^−^-dependent reversal potential changes seen in OsHKT1;1-V2-expressing oocytes (Figure 4C,D, Supplementary Figure S6C,D), water-injected controls showed only small inward and outward currents that did not change with external Cl^−^ concentration (Supplementary Figure S4B). Second, Cl^−^ accumulation assays demonstrated that water-injected oocytes consistently showed minimal Cl^−^ accumulation under all tested conditions, and OsHKT1;1-V2-expressing oocytes showed an increase in Cl^−^ accumulation (Figure 6). Note, however, that we cannot exclude the possibility that the OsHKT1;1-V2 variant interacts with other membrane proteins to form a minimal Cl^−^-selective channel in oocytes. Thus, further mechanistic research will be needed. Approaches such as expressing dominant-negative OsHKT1;1-V2 mutants, testing OsHKT1;1-V2 multimerization biochemically, and reconstituting purified OsHKT1;1-V2 into liposomes or artificial membranes would clarify whether OsHKT1;1-V2 exhibits an inherently channel-forming feature by itself.

Although some amino acid substitutions were detected in the variants of *OsHKT1;1* between Pokkali and Nipponbare (Supplementary Figure S5), the lineup of *OsHKT1;1* transcripts was identical between the two cultivars [8]. In addition, OsHKT1;1-FL and other OsHKT1;1 variants derived from Nipponbare exhibited transport profiles similar to those of OsHKT1;1 transporters from Pokkali (Figure 8) [8]. Moreover, the similarity in the substrate selectivity of the OsHKT1;1-V2 variants from both rice varieties suggested that the function of OsHKT1;1-V2 in ion homeostasis might be conserved across different genetic backgrounds (Figure 4 and Figure 5, Supplementary Figure S6).

It has been reported that the transcript levels of *OsHKT1;1* in shoots increased significantly, showing a 3- to 5-fold rise after 12 h of salt treatment, whereas no upregulation was observed in roots [22]. In contrast, *HvHKT1;1* was upregulated in roots under salinity stress, with relatively lower expression levels detected in leaves and sheaths [41]. Similarly, the transcript levels of *HKT1;1* have been analyzed in other plant species, in which the expression level was found to be increased in response to salt stress in both shoots and roots, or only in roots [58,59,60]. However, previous reports have only quantified full-length mRNA and have not considered variants. Absolute quantification of *OsHKT1;1* transcripts in the present and previous studies demonstrated that the expression levels varied among variants and between the cultivars. Nipponbare exhibited higher and more stable expression of *OsHKT1;1-FL* under salt stress (Figure 9). As *OsHKT1;1* has been proposed to be a salt-tolerant determinant in rice [22,23,24], it is intriguing that salt-tolerant Pokkali did not increase *OsHKT1;1* transcripts and maintained a lower level of expression during salt stress compared with those in salt-sensitive Nipponbare (Figure 9). These results indicate that *OsHKT1;1* may function to maintain basal ion homeostasis in Pokkali under normal condition and have a limited role in Pokkali under salt stress, although *OsHKT1;1* in a salt-tolerant *indica* rice cultivar Zhenshan 2 was found to play a more essential role in the mechanism of salt tolerance [23]. In Pokkali, other specific transporters/channels, including OsHKT1;5 [2], may have significant functions under salt stress.

Overall, these results highlight the unexpected characteristics of the *OsHKT1;1* splicing variant OsHKT1;1-V2 in rice, which was suggested to encode a possible Cl^−^ channel. However, the expression of *OsHKT1;1-V2* was lower than that of *OsHKT1;1-FL* in both Pokkali and Nipponbare. However, important questions remain to be elucidated: (i) Does OsHKT1;1-V2 function as a Cl^−^ channel in rice? If so, (ii) does OsHKT1;1-V2 contribute to Cl^−^ homeostasis and salt tolerance? (iii) Does the differential regulation in the expression of *OsHKT1;1-FL* and *OsHKT1;1-V2* between Nipponbare and Pokkali reflect the mechanism of salt tolerance in rice? In addition, further studies are needed to explore the regulatory mechanisms behind the splicing of *OsHKT1;1* and to investigate whether some of the variants other than OsHKT1;1-V2 could also contribute to ion homeostasis in rice plants, including upon salt stress.

## 4. Materials and Methods

### 4.1. Plant Material and Growth Conditions

Seeds of Nipponbare (*Oryza sativa* L. ssp. *japonica*), a salt-sensitive rice cultivar, were sterilized, germinated, and grown hydroponically as described previously [8]. Total RNA was extracted from 14-day-old plants.

### 4.2. Extraction of RNA, and cDNA Synthesis

Total RNA was extracted, and its quality and integrity were assessed before synthesizing cDNA, following a previously described protocol [8]. The *OsHKT1;1* cDNAs were then amplified using *OsHKT1;1* cloning primers and subsequently inserted into a pCR4 topo vector (Invitrogen, Carlsbad, CA, USA).

### 4.3. Preparation of cRNA for X. laevis Oocytes Heterologous Expression

Full-length *OsHKT1:1* (*OsHKT1;1-FL*) and a variant (*OsHKT1;1-V2*) cRNAs from Pokkali (*Oryza sativa* L. ssp. *indica*) have already been prepared [8]. The cDNAs of *OsHKT1;1* from Nipponbare (*Oryza sativa* L. ssp. *japonica*) were cloned into a pXβG vector, and capped RNA (cRNA) synthesis was performed according to the procedure outlined by Imran et al. [8]. Oocytes were collected and injected with cRNA at concentrations of 50 ng/50 nL or 2.5 ng/50 nL, or with 50 nL of nuclease-free water as a negative control. The oocytes were then incubated at 18 °C, unless otherwise mentioned, in a modified Barth’s solution (MBS) until electrophysiological measurements were conducted as described [61].

### 4.4. Electrophysiology

Oocyte currents were measured using the two-electrode voltage clamp (TEVC) technique, conducted 1 d after cRNA injection, unless otherwise mentioned. TEVC recordings and subsequent data analysis were performed using an Axoclamp 900A amplifier (Molecular Devices, San Jose, CA, USA), Axon Instruments Digidata 1440A (Molecular Devices, San Jose, CA, USA), and pCLAMP 10 software (Molecular Devices, San Jose, CA, USA). The bath solutions were prepared with a baseline concentration of 1.8 mM MgCl_2_, 1.8 mM CaCl_2_, and 10 mM HEPES at pH 7.5 with Tris, and chloride salts of the monovalent cations were used. Gluconic acid magnesium (II) salt or D-mannitol was added as needed to maintain the osmolality of the external solutions between 200 and 220 mOsm in all measurements. To obtain current–voltage relationships, voltage steps (2 s) were applied from +30 to −120 mV in 15 mV decrements unless otherwise mentioned.

### 4.5. Determination of Cl^−^ Content in X. laevis Oocytes

Oocytes were injected with 50 nL of water or cRNA of *OsHKT1;1-FL* (2.5 ng) and *OsHKT1;1-V2* (2.5 ng and 25 ng), then incubated in 1× MBS medium for 24 h (*OsHKT1;1-FL*) or 4 h (*OsHKT1;1-V2*) at 18 °C. For the measurement of Cl^−^ accumulation, the oocytes were transferred to 1 mL of Na-gluconate or choline-Cl solutions. The Na-gluconate solution contained 96 mM Na gluconate, 1.8 mM Ca gluconate, 1.8 mM Mg gluconate, 1.8 mM mannitol, and 10 mM HEPES (pH 7.5 with Tris). The choline-Cl solution contained 96 mM choline Cl, 1.8 mM Ca gluconate, 1.8 mM Mg gluconate, 1.8 mM mannitol, and 10 mM HEPES (pH 7.5 with Tris). After a 6 h incubation at room temperature, the oocytes were washed three times with isotonic Ca-gluconate solution and then placed in a 1.5 mL tube to homogenize in Milli-Q water. Cell debris was removed by centrifugation. The supernatant was collected, diluted, and used for ion analysis using an anion chromatography system.

### 4.6. Subcellular Localization Analysis Using Arabidopsis Protoplasts

Subcellular localization analysis of OsHKT1;1-FL and OsHKT1;1-V2 proteins was conducted in Arabidopsis leaf protoplasts [35], which were transfected with the modified pTH2 vector [62] containing OsHKT1;1-FL or OsHKT1;1-V2 fused with GFP at the N-terminus under the control of the CaMV 35S promoter. Transfection was performed using the polyethylene glycol (PEG 4000) method, as described by Yoo et al. [63] and Sasaki et al. [64]. Protoplasts suspended in the re-suspension solution (0.5 M mannitol, 20 mM KCl, and 4 mM MES, pH 5.7) were incubated in the dark at 24 °C for 16 h [65]. The plasma membrane was visualized using FM4-64 (16 μM, 5 min). Images of the protoplasts were captured using an Olympus FluoView 1000 Confocal Microscope (Olympus Corporation, Hachioji, Tokyo, Japan). GFP was detected using 473 nm excitation with a 490–525 nm bandpass filter, whereas FM4-64 and chlorophyll were observed with 543 nm excitation and a 560–620 nm bandpass filter.

### 4.7. Subcellular Localization in Oocytes by Immunocytochemistry

Subcellular localization in oocytes was analyzed using OsHKT1;1-FL and OsHKT1;1-V2 with a FLAG tag (DYKDDDDK) at their N-termini. cRNAs were synthesized using an oocyte expression vector, pXβG vector, including O*sHKT1;1-FL* or *OsHKT1;1-V2* with the sequence of FLAG tag, according to the procedure outlined by Imran et al. [8]. Immunohistochemical analysis was performed as described by Shibasaka et al. [66]. A volume of 50 ng/50 nl for OsHKT1;1-FL and 2.5 ng/50 nl for OsHKT1;1-V2 cRNAs or RNase-free water as a negative control was injected into the oocyte and incubated at 18 °C for 24 h (OsHKT1;1-FL) or 4 h (OsHKT1;1-V2). Subsequently, the samples were fixed for 3 h in 4% (*w*/*v*) formaldehyde solution (pH 7.4) and embedded in 5% agarose. Sections of 100 μm thickness were prepared using a micro slicer (Doshin EM, Osaka, Japan) and subjected to blocking in a buffer containing 50 mM Tris (pH 8.0), 150 mM NaCl, 0.1% Tween 20, and 3% BSA for 1 h at 25 °C. Immunolabeling was performed using primary antibodies, including rat anti-FLAG (raised against the synthetic peptide CDYKDDDDK, Invitrogen, Carlsbad, CA, USA), applied overnight at 4 °C. After three washes, the samples were incubated for another hour with a secondary antibody (Alexa Fluor 488-conjugated goat anti-rabbit IgG, Invitrogen, Carlsbad, CA, USA). After two washes with TBS-T (50 mM Tris, pH 8.0, 150 mM NaCl, 0.1% Tween 20) and a final wash with TBS (50 mM Tris, pH 8.0, 150 mM NaCl), fluorescent signals were observed using a fluorescence microscope (BZ-X700, Keyence, Osaka, Japan).

### 4.8. Functional Characterization of OsHKT1;1-FL and OsHKT1;1-V2 in Yeast

The CDSs of *OsHKT1;1-FL* and *OsHKT1;1-V2* were cloned into the pYES2 vector and transformed into the well-known Na^+^-sensitive yeast strain G19, which lacks Na^+^ efflux transporters ENA1-4 [67,68]. Positive transformants were selected on Ura-selective medium plates [0.67% (*w*/*v*) yeast nitrogen base without amino acids, 0.077% (*w*/*v*) dropout mix-Ura, 2% (*w*/*v*) glucose, and 2% (*w*/*v*) agar]. For the yeast growth test, all transformed yeasts were cultured overnight at 30 °C in SC-Ura medium until the OD_600_ reached 1.0, and 10-fold serially diluted cultures were incubated on Ura-induced medium [0.67% (*w*/*v*) yeast nitrogen base without amino acids, 0.077% (*w*/*v*) dropout mix-Ura, 2% (*w*/*v*) galactose, 1% (*w*/*v*) raffinose, and 2% (*w*/*v*) agar] plates containing 50 mM NaCl. The plates were incubated at 30 °C for 4 days, and yeast cell growth was recorded.

### 4.9. Determination of Ion Contents in Yeast Cells

To quantify intracellular Na^+^, each transgenic G19 line was grown in liquid synthetic complete (SC) medium supplemented with or without 50 mM NaCl and galactose at 30 °C. Prior to the main culture, each transformant was cultured in a glass test tube filled with 5 mL SC-Ura glucose medium for overnight at 30 °C. 0.1 mL of each full growth-culture solution was inoculated in a 300 mL Erlenmeyer flask filled with 100 mL SC-Ura galactose medium. After incubation for 18 h (control) and 21 h (NaCl) at 30 °C at 130 rpm, each line was collected by swing-bucket centrifugation using 50 mL centrifuge tubes and then washed by 100 mL of sterilized ultrapure water. After 3 time washes, 10 mL of the washed culture solution was devided in a 15 mL centrifuge tube, and then samples were collected by swing-bucket centrifugation. All harvested samples were dried at 60 °C for 2 days. The dried samples were weighed and digested by 1 mL of ultrapure nitric acid for 24 h. Digests were subsequently heated at 95 °C for 15 min, which was repeated two times. Sodium (Na^+^) concentrations were quantified by inductively coupled plasma–mass spectrometry (ICP-MS).

### 4.10. Expression Analysis

Total RNA extraction, first-strand cDNA synthesis, and absolute quantification of gene expression using specific cDNAs standards were performed as previously described [8]. Total RNA was treated with DNase to remove contaminating genomic DNA (gDNA).

### 4.11. Statistical Analyses

Statistical analyses were performed using IBM SPSS Statistics (version 25). Significant differences were identified using a *t*-test (*** *p* < 0.001) for Figure 7 and a one-way ANOVA, and Tukey’s HSD test (*p* < 0.05) for Figure 6.

## Data Availability

The data supporting the findings of this study are available from the corresponding authors upon reasonable request.

## Supplementary Materials

The following supporting information can be downloaded at: https://www.mdpi.com/article/10.3390/ijms27031178/s1.

## Abbreviations

The following abbreviations are used in this manuscript:

cDNA	Complementary DNA
cRNA	Capped RNA
gDNA	Genomic DNA
GFP	Green fluorescence protein
HKT	High-Affinity Potassium Transporter
mRNA	Messenger RNA
SOS	Salt Overly Sensitive
TEVC	Two-electrode voltage clamp
