# Supplementary material for: Chloride-Transporting OsHKT1;1 Splice Variants and Their Expression Profiles Under Salinity Stress in Rice
Source: Int J Mol Sci. 2026 Jan 23;27(3):1178. doi: 10.3390/ijms27031178 (PMC12898316; doi:10.3390/ijms27031178)
Supplement: Supplementary file 1 [file ijms-27-01178-s001.zip › ijms-4037651-supplementary.pdf]

Chloride-Transporting OsHKT1;1 Splice Variants and Their Expression Profiles Under Salinity Stress in Rice

Shahin Imran, Shuntaro Ono, Rie Horie, Maki Katsuhara and Tomoaki Horie

Supplementary Materials: Figure S1. Amino acid sequence alignment of OsHKT1;1-FL and OsHKT1;1-V2.; **Figure S2.** Current-voltage relationships in *X. laevis* oocytes expressing OsHKT1;1-FL (Pokkali-derived); **Figure S3**. Current-voltage relationships of oocytes expressing OsHKT1;1-V2 or the oocytes injected with water as a negative control; **Figure S4**. Current-voltage relationships of oocytes injected with water, obtained under various concentrations of Na gluconate or Choline Cl; **Figure S5**. Comparisons of amino acid sequences of OsHKT1;1-FL and OsHKT1;1 variants between Nipponbare and Pokkali; **Figure S6.** Chloride concentration dependency of OsHKT1;1-FL and OsHKT1;1-V2, derived from Nipponbare and expressed in *X. laevis* oocytes; **Figure S7**. Sodium concentration dependency of OsHKT1;1-FL.

A

```
OsHKT1;1-FL       1 MHPPSLVLDTLKRIKLYIAMKLLLPNSEVLRIYWEKAQHLCGFLSMKLISRARCVASSVK 60
OsHKT1;1-V2(1)    1 MHPPSLVLDTLKRIKLYIAMKLLLPNSEVLRIYWEKAQHLCGFLSMKLISRARCV----- 55

OsHKT1;1-FL      61 QSYSFLVCKSNPLVVQLVYFVIISFAGFLALKNLKPQGKPGPKDLDLLFTSVSTLTVSSM 120
OsHKT1;1-V2(1)   56 --------------------------------NLKPQGKPGPKDLDLLFTSVSTLTVSSM 83
                                                         GKxGPxx pattern

OsHKT1;1-FL     121 ATVEMEDLSDRQLWVLILLMLMGGEVFTSMLGLYFNNANANRNENSQRSLPSISLDIESN 180
OsHKT1;1-V2(1)   84 ATVEMEDLSDRQLWVLILLMLMGGEVFTSMLGLYFNNANANRNENSQRSLPSISLDIESN 143
                                 TM of -V2

OsHKT1;1-FL     181 SPANNGDHKITECGQSEETMSQNQVQQNKSITYNPCAVLVRIVTGYFVATVISSSVIIII 240
OsHKT1;1-V2(1)  144 SPANNGDHKITECGQSEETMSQNQMQEMY------------------------------- 172

OsHKT1;1-FL     241 YFWIDSDARNVLKSKEISMYTFCIFTAVSSFANCGFTPLNSNMQPFRKNWVLLLLVIPQI 300
OsHKT1;1-V2(1)  172 ------------------------------------------------------------ 172

OsHKT1;1-FL     301 LAGNTLFSPLLRLCVWVLGKVSGKAEYAYILQHPGETGYKHLHVRRNSVYIVLSVTGLIL 360
OsHKT1;1-V2(1)  172 ------------------------------------------------------------ 172

OsHKT1;1-FL     361 LQVMFICSFEWNSESLEGMNWLQKLVGLLFQSVNTRQAGESILDISTLSPSTLLLFAVVM 420
OsHKT1;1-V2(1)  172 ------------------------------------------------------------ 172

OsHKT1;1-FL     421 YLPSDASFLTANADNQPLTDKKTNSISRALWRNFTVNKLSCLAMFTFLACITERKSISSD 480
OsHKT1;1-V2(1)  172 ------------------------------------------------------------ 172

OsHKT1;1-FL     481 PLNFNIFSIVFEIISAFGNVGYSLGYSCQKLLKPDATCKDASYGFVGRWTEEGKLIVILV 540
OsHKT1;1-V2(1)  172 ------------------------------------------------------------ 172

OsHKT1;1-FL     541 MFLGRLKEFILK                                                 552
OsHKT1;1-V2(1)  172 ------------                                                 172
```

B

```
OsHKT1;1-FL       1 MHPPSLVLDTLKRIKLYIAMKLLLPNSEVLRIYWEKAQHLCGFLSMKLISRARCVASSVK 60
OsHKT1;1-V2(2)    0 ------------------------------------------------------------ 0

OsHKT1;1-FL      61 QSYSFLVCKSNPLVVQLVYFVIISFAGFLALKNLKPQGKPGPKDLDLLFTSVSTLTVSSM 120
OsHKT1;1-V2(2)    0 ------------------------------------------------------------ 0

OsHKT1;1-FL     121 ATVEMEDLSDRQLWVLILLMLMGGEVFTSMLGLYFNNANANRNENSQRSLPSISLDIESN 180
OsHKT1;1-V2(2)    0 ------------------------------------------------------------ 0

OsHKT1;1-FL     181 SPANNGDHKITECGQSEETMSQNQVQQNKSITYNPCAVLVRIVTGYFVATVISSSVIIII 240
OsHKT1;1-V2(2)    1 ------------------------------------------------------MWPIRR 6

OsHKT1;1-FL     241 YFWIDSDARNVLKSKEISMYTFCIFTAVSSFANCGFTPLNSNMQPFRKNWVLLLLVIPQI 300
OsHKT1;1-V2(2)    7 NYVAKPDARNVLKSKEISMYTFCIFTAVSSFANCGFTPLNSNMQPFRKNWVLLLLVIPQI 66

OsHKT1;1-FL     301 LAGNTLFSPLLRLCVWVLGKVSGKAEYAYILQHPGETGYKHLHVRRNSVYIVLSVTGLIL 360
OsHKT1;1-V2(2)   67 LAGNTLFSPLLRLCVWVLGKVSGKAEYAYILQHPGETGYKHLHVRRNSVYIVLSVTGLIL 126

OsHKT1;1-FL     361 LQVMFICSFEWNSESLEGMNWLQKLVGLLFQSVNTRQAGESILDISTLSPSTLLLFAVVM 420
OsHKT1;1-V2(2)  127 PQVMFICSFEWNSESLEGMNWLQKLVGLLFQSVNTRQAGESILDISTLSPSTLLLFAVVM 186

OsHKT1;1-FL     421 YLPSDASFLTANADNQPLTDKKTNSISRALWRNFTVNKLSCLAMFTFLACITERKSISSD 480
OsHKT1;1-V2(2)  187 YLPSDASFLTANADNQPLTDKKTNSISRALWRNFTVNKLSCLAMFTFLACITERKSISSD 246

OsHKT1;1-FL     481 PLNFNIFSIVFEIISAFGNVGYSLGYSCQKLLKPDATCKDASYGFVGRWTEEGKLIVILV 540
OsHKT1;1-V2(2)  247 PLNFNIFSIVFEIISAFGNVGYSLGYSCQKLLKPDATCKDASYGFVGRWTEEGKLIVILV 306

OsHKT1;1-FL     541 MFLGRLKEFILK                                                 552
OsHKT1;1-V2(2)  307 MFLGRLKEFILK                                                 318
```

Supplementary Figure S1. Amino acid sequence alignment of OsHKT1;1-FL and OsHKT1;1-V2. (**A**) OsHKT1;1-FL vs. OsHKT1;1-V2(1). (**B**) OsHKT1;1-FL vs. OsHKT1;1-V2(2). GENETYX ver. 16 was used for comparisons.

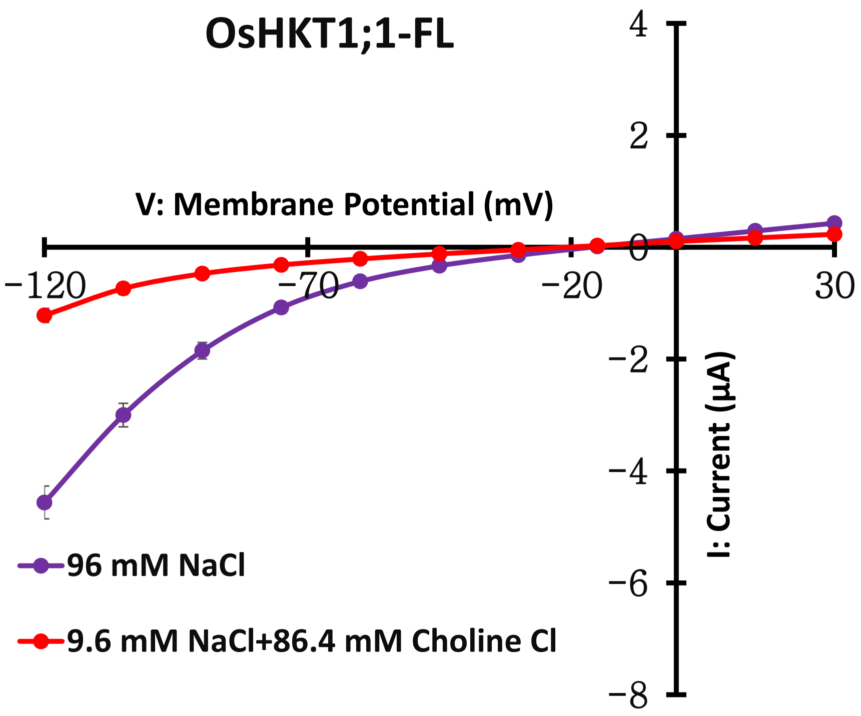

Supplementary Figure S2. Current-voltage relationships in *X. laevis* oocytes expressing OsHKT1;1-FL (Pokkali-derived). The external solutions used were 96 mM NaCl or 9.6 mM NaCl + 86.4 mM choline chloride. All external solutions contain, as background elements, 1.8 mM $CaCl_2$, 1.8 mM $MgCl_2$, 1.8 mM mannitol, and 10 mM HEPES (pH 7.5 with Tris). Oocytes were injected with 50 ng cRNAs/50 nL and incubated for 18 h at 18 °C before TEVC measurements. Data are presented as mean ± SE, n = 8, from two independent experiments performed with different batches of oocytes.

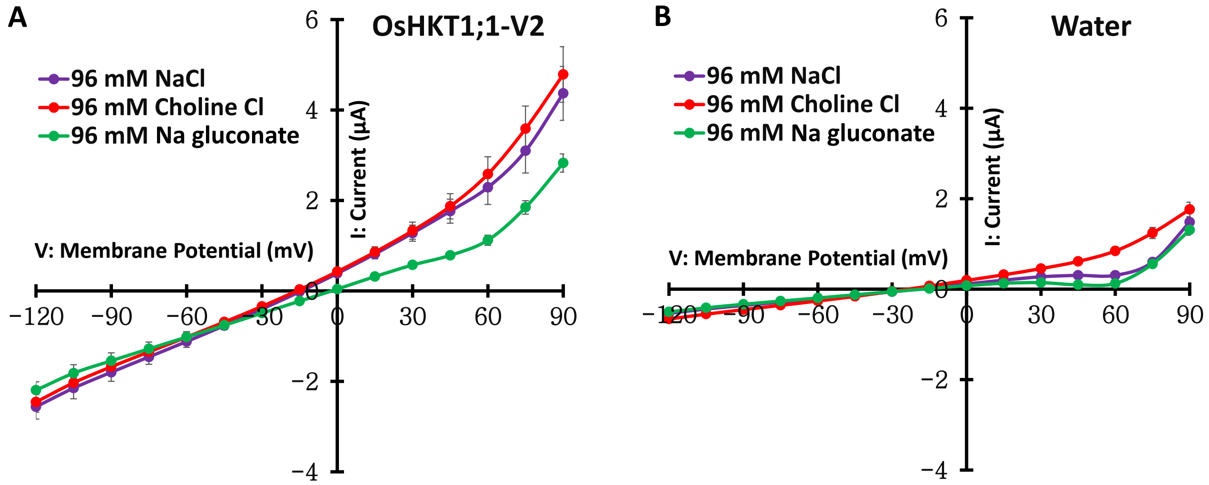

Supplementary Figure S3. Current-voltage relationships of oocytes expressing OsHKT1;1-V2 (**A**) or oocytes injected with water (**B**) as a negative control. The external solutions included 96 mM NaCl, 96 mM Na gluconate, or 96 mM Choline Cl. The 96 mM NaCl solution contained background elements such as 1.8 mM $CaCl_2$, 1.8 mM $MgCl_2$, 1.8 mM mannitol, and 10 mM HEPES (pH 7.5 with Tris). The 96 mM Na gluconate and 96 mM choline chloride solutions contained background elements such as 1.8 mM Ca gluconate, 1.8 mM Mg gluconate, 1.8 mM mannitol, and 10 mM HEPES (pH 7.5 with Tris). Data are presented as mean ± SE, n = 10–16, from two independent experiments using different batches of oocytes.

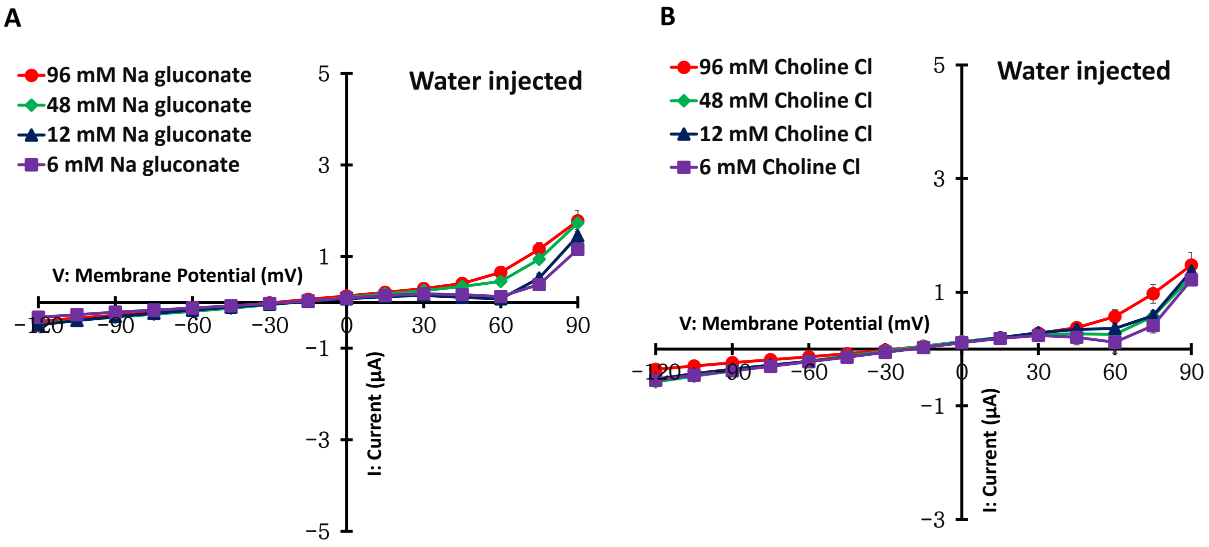

Supplementary Figure S4. Current-voltage relationships of oocytes injected with water, obtained under various concentrations of Na gluconate (**A**) or Choline Cl (**B**). Na gluconate solutions contained basic components as 1.8 mM Ca gluconate, 1.8 mM Mg gluconate, 1.8 mM mannitol, and 10 mM HEPES, and the pH was adjusted to 7.5 using Tris. Choline Cl solutions contained basic components as 1.8 mM $CaCl_2$, 1.8 mM $MgCl_2$, 1.8 mM mannitol, and 10 mM HEPES, and the pH was adjusted to 7.5 with Tris. The 48-, 12-, and 6-mM sodium gluconate and choline chloride solutions contained 38-, 63-, and 68-mM Mg gluconate, respectively. Data are presented as means ± SE, *n* = 6–8, from two independent experiments performed on different oocyte batches.

A

```
OsHKT1;1-FL (N)   1 MHPPSLVLDTLKRIKLYIAMKLLLPNSEVPRIYWEKAQHLCGFLSMKLISRARCVASSVK 60
OsHKT1;1-FL (P)   1 MHPPSLVLDTLKRIKLYIAMKLLLPNSEVLRIYWEKAQHLCGFLSMKLISRARCVASSVK 60

OsHKT1;1-FL (N)  61 QSYSFLVCKSNPLVVQLVYFVIISFAGFLALKNLKPQGKPGPKDLDLLFTSVSTLTVSSM 120
OsHKT1;1-FL (P)  61 QSYSFLVCKSNPLVVQLVYFVIISFAGFLALKNLKPQGKPGPKDLDLLFTSVSTLTVSSM 120

OsHKT1;1-FL (N) 121 ATVEMEDLSDRQLWVLILLMLMGGEVFTSMLGLYFNNANANRNENSQRSLPSISLDIEFN 180
OsHKT1;1-FL (P) 121 ATVEMEDLSDRQLWVLILLMLMGGEVFTSMLGLYFNNANANRNENSQRSLPSISLDIESN 180

OsHKT1;1-FL (N) 181 SPANNGDHKITECGQSEETMSQNQVQQNKSITYNPCAVLVRIVTGYFVATVISSSVIIII 240
OsHKT1;1-FL (P) 181 SPANNGDHKITECGQSEETMSQNQVQQNKSITYNPCAVLVRIVTGYFVATVISSSVIIII 240

OsHKT1;1-FL (N) 241 YFWIDSDARNVLKSKEINMYTFCIFTAVSSFANCGFTPLNSNMQPFRKNWVLLLLVIPQI 300
OsHKT1;1-FL (P) 241 YFWIDSDARNVLKSKEISMYTFCIFTAVSSFANCGFTPLNSNMQPFRKNWVLLLLVIPQI 300

OsHKT1;1-FL (N) 301 LAGNTLFSPLLRLCVWVLGKVSGKAEYAYILQHPGETGYKHLHVRRNSVYIVLSVTGLIL 360
OsHKT1;1-FL (P) 301 LAGNTLFSPLLRLCVWVLGKVSGKAEYAYILQHPGETGYKHLHVRRNSVYIVLSVTGLIL 360

OsHKT1;1-FL (N) 361 LQVMFICSFEWNSESLEGMNWLQKLVGLLFQSVNTRQAGESILDISTLSPSTLLLFAVVM 420
OsHKT1;1-FL (P) 361 LQVMFICSFEWNSESLEGMNWLQKLVGLLFQSVNTRQAGESILDISTLSPSTLLLFAVVM 420

OsHKT1;1-FL (N) 421 YLPSDASFLTANADNQPLTDKKTNSISRALWRNFTVNKLSCLAMFTFLACITERKSISSD 480
OsHKT1;1-FL (P) 421 YLPSDASFLTANADNQPLTDKKTNSISRALWRNFTVNKLSCLAMFTFLACITERKSISSD 480

OsHKT1;1-FL (N) 481 PLNFNIFSIVFEIISAFGNVGYSLGYSCQKLLKPDTTCKDASYGFVGRWTEEGKLIVILV 540
OsHKT1;1-FL (P) 481 PLNFNIFSIVFEIISAFGNVGYSLGYSCQKLLKPDATCKDASYGFVGRWTEEGKLIVILV 540

OsHKT1;1-FL (N) 541 MFLGRLKEFILK                                                 552
OsHKT1;1-FL (P) 541 MFLGRLKEFILK                                                 552
```

B

```
OsHKT1;1-V1 (N)   1 MHPPSLVLDTLKRIKLYIAMKLLLPNSEVLRIYWEKAQHLCGFLSMKLISRARCVNLKPQ 60
OsHKT1;1-V1 (P)   1 MHPPSLVLDTLKRIKLYIAMKLLLPNSEVLRIYWEKAQHLCGFLSMKLISRARCVNLKPQ 60

OsHKT1;1-V1 (N)  61 GKPGPKDLDLLFTSVSTLTVSSMATVEMEDLSDRQLWVLILLMLMGGEVFTSMLGLYFNN 120
OsHKT1;1-V1 (P)  61 GKPGPKDLDLLFTSVSTLTVSSMATVEMEDLSDRQLWVLILLMLMGGEVFTSMLGLYFNN 120

OsHKT1;1-V1 (N) 121 ANANRNENSQRSLPSISLDIESNSPANNGDHKITECGQSEETMSQNQVQQNKSITYNPCA 180
OsHKT1;1-V1 (P) 121 ANANRNENSQRSLPSISLDIESNSPANNGDHKITECGQSEETMSQNQVQQNKSITYNPCA 180

OsHKT1;1-V1 (N) 181 ALVRIVTGYFVATVISSSVIIIIYFWIDSDAGNVLKSKEISMYTFCIFTAVSSFANCGFT 240
OsHKT1;1-V1 (P) 181 ALVRIVTGYFVATVISSSVIIIIYFWIDSDARNVLKSKEISMYTFCIFTAVSSFANCGFT 240

OsHKT1;1-V1 (N) 241 SLNSNMQPFRKNWVLLLLVIPQILAGNTLFSPLLRLCVWVLGKVSGKAEYAYILQHPGET 300
OsHKT1;1-V1 (P) 241 PLNSNMQPFRKNWVLLLLVIPQILAGNTLFSPLLRLCVWVLGKVSGKAEYAYILQHPGET 300

OsHKT1;1-V1 (N) 301 GYKHLHVRRNSVYIVLSVTGLILLQVMFICSFEWNSESLEGMNWLQKLVGLLFQSVNTRQ 360
OsHKT1;1-V1 (P) 301 GYKHLHVRRNSVYIVLSVTGLILLQVMFICSFEWNSESLEGMNWLQKLVGLLFQSVNTRQ 360

OsHKT1;1-V1 (N) 361 AGESILDISTLSPSTLLLFAVVMYLPSDASFLTANADNQPLTDKKTNSISRALWRNFTVN 420
OsHKT1;1-V1 (P) 361 AGESILDISTLSPSTLLLFAVVMYLPSDASFLTANADNQPLTDKKTNSISRALWRNFTVN 420

OsHKT1;1-V1 (N) 421 KLSCLAMFTFLACITERKSISSDPLNFNIFSIVFEIISAFGNVGYSLGYSCQKLLKPDAT 480
OsHKT1;1-V1 (P) 421 KPSCLAMFTFLACITERKSISSDPLNFNIFSIVFEIISAFGNVGYSLGYSCQKLLKPDAT 480

OsHKT1;1-V1 (N) 481 CKDASYGFVGRWTEEGKLIVILVMFLGRLKEFILK                          515
OsHKT1;1-V1 (P) 481 CKDASYGSVGRWTEEGKLIVILVMFLGRLKEFILK                          515
```

C

```
OsHKT1;1-V2 (N)   1 MHPPSLVLDTLKRIKLYIAMKLLLPNSEVPRIYWEKAQHLCGFLSMKLISRARCVNLKPQ 60
OsHKT1;1-V2 (P)   1 MHPPSLVLDTLKRIKLYIAMKLLLPNSEVLRIYWEKAQHLCGFLSMKLISRARCVNLKPQ 60

OsHKT1;1-V2 (N)  61 GKPGPKDLDLLFTSVSTLTVSSMATVEMEDLSDRQLWVLILLMLMGGEVFTSMLGLYFNN 120
OsHKT1;1-V2 (P)  61 GKPGPKDLDLLFTSVSTLTVSSMATVEMEDLSDRQLWVLILLMLMGGEVFTSMLGLYFNN 120

OsHKT1;1-V2 (N) 121 ANANRNENSQRSLPSISLDIEFNSPANNGDHKITECGQSEETMSQNQMQEMY         172
OsHKT1;1-V2 (P) 121 ANANRNENSQRSLPSISLDIESNSPANNGDHKITECGQSEETMSQNQMQEMY         172
```

```
D   OsHKT1;1-V3  (N)    1  MHPPSLVLDTLKRIKLYIAMKLLLPNSEVPRIYWEKAQHLCGFLSMKLISRARCVASSVK  60
    OsHKT1;1-V3  (P)    1  MHPPSLVLDTLKRIKLYIAMKLLLPNSEVLRIYWEKAQHLCGFLSMKLISRARCVASSVK  60

    OsHKT1;1-V3  (N)   61  QSYSFLVCKSNPLVVQLVYFVIISFAGFLALKNLKPQGKPGPKDLDLLFTSVSTLTVSSM  120
    OsHKT1;1-V3  (P)   61  QSYSFLVCKSNPLVVQLVYFVIISFAGFLALKNLKPQGKPGPKDLDLLFTSVSTLTVSSM  120

    OsHKT1;1-V3  (N)  121  ATVEMEDLSDRQLWVLILLMLMGGEVFTSMLGLYFNNANANRNENSQRSLPSISLDIEFN  180
    OsHKT1;1-V3  (P)  121  ATVEMEDLSDRQLWVLILLMLMGGEVFTSMLGLYFNNANANRNENSQRSLPSISLDIESN  180

    OsHKT1;1-V3  (N)  181  SPANNGDHKITECGQSEETMSQNQMQEMY                                 209
    OsHKT1;1-V3  (P)  181  SPANNGDHKITECGQSEETMSQNQMQEMY                                 209

E   OsHKT1;1-V4  (N)    1  MHPPSLVLDTLKRIKLYIAMKLLLPNSEVPRIYWEKAQHLCGFLSMKLISRARCVASSVK  60
    OsHKT1;1-V4  (P)    1  MHPPSLVLDTLKRIKLYIAMKLLLPNSEVLRIYWEKAQHLCGFLSMKLISRARCVASSVK  60

    OsHKT1;1-V4  (N)   61  QSYSFLVCKSNPLVVQLVYFVIISFAGFLALKNLKPQGKPGPKDLDLLFTSVSTLTVSSM  120
    OsHKT1;1-V4  (P)   61  QSYSFLVCKSNPLVVQLVYFVIISFAGFLALKNLKPQGKPGPKDLDLLFTSVSTLTVSSM  120

    OsHKT1;1-V4  (N)  121  ATVEMEDLSDRQLWVLILLMLMGGEVFTSMLGLYFNNANANRNENSQRSLPSISLDIEFN  180
    OsHKT1;1-V4  (P)  121  ATVEMEDLSDRQLWVLILLMLMGGEVFTSMLGLYFNNANANRNENSQRSLPSISLDIESN  180

    OsHKT1;1-V4  (N)  181  SPANNGDHKITECGQSEETMSQNQVPSFRCFISHCQC                         217
    OsHKT1;1-V4  (P)  181  SPANNGDHKITECGQSEETMSQNQVPSFRCFISHCQC                         217

F   OsHKT1;1-V5  (N)    1  MHPPSLVLDTLKRIKLYIAMKLLLPNSEVPRIYWEKAQHLCGFLSMKLISRARCVNLKPQ  60
    OsHKT1;1-V5  (P)    1  MHPPSLVLDTLKRIKLYIAMKLLLPNSEVLRIYWEKAQHLCGFLSMKLISRARCVNLKPQ  60

    OsHKT1;1-V5  (N)   61  GKPGPKDLDLLFTSVSTLTVSSMATVEMEDLSDRQLWVLILLMLMGGEVFTSMLGLYFNN  120
    OsHKT1;1-V5  (P)   61  GKPGPKDLDLLFTSVSTLTVSSMATVEMEDLSDRQLWVLILLMLMGGEVFTSMLGLYFNN  120

    OsHKT1;1-V5  (N)  121  ADANRNENSQRSLPSISLDIEFNSPANNGDHKITECGQSEETMSQNQVPSFRCFISHCQC  180
    OsHKT1;1-V5  (P)  121  ANANRNENSQRSLPSISLDIESNSPANNGDHKITECGQSEETMSQNQVPSFRCFISHCQC  180

G   OsHKT1;1-V6  (N)    1  MHPPSLVLDTLKRIKLYIAMKLLLPNSEVLRIYWEKAQHLCGFLSMKLISRARCVASSVK  60
    OsHKT1;1-V6  (P)    1  MHPPSLVLDTLKRIKLYIAMKLLLPNSEVLRIYWEKAQHLCGFLSMKLISRARCVASSVK  60

    OsHKT1;1-V6  (N)   61  QSYSFLVCKSDPLVVQLVYFVIISFAGFLALKNLKPQGKPGPKDLDLLFTSVSTLTVSSM  120
    OsHKT1;1-V6  (P)   61  QSYSFLVCKSNPLVVQLVYFVIISFAGFLALKNLKPQGKPGPKDLDLLFTSVSTLTVSSM  120

    OsHKT1;1-V6  (N)  121  ATVEMEDLSDRQLWVLILLMLMGGEVFTSMLGLYFNIFSIVFEIISAFGNVGYSLGYSCQ  180
    OsHKT1;1-V6  (P)  121  ATVEMEDLSDRQLWVLILLMLMGGEVFTSMLGLYFNIFSIVFEIISAFGNVGYSLGYSCQ  180

    OsHKT1;1-V6  (N)  181  KLLKPDATCKDASYGFVGRWTEEGKLIVILVMFLGRLKEFILK                   223
    OsHKT1;1-V6  (P)  181  KLLKPDATCKDASYGFVGRWTEEGKLIVILVMFLGRLKEFILK                   223

H   OsHKT1;1-V7  (N)    1  MHPPSLVLDTLKRIKLYIAMKLLLPNSEVLRIYWEKAQHLCGFLSMKLISRARCVNLKPQ  60
    OsHKT1;1-V7  (P)    1  MHPPSLVLDTLKRIKLYIAMKLLFPNSEVLRIYWEKAQHLCGFLSMKLISRARCVNLKPQ  60

    OsHKT1;1-V7  (N)   61  GKPGPKDLDLLFTSVSTLTVSSMATVEMEDLSDRQLWVLILLMLMGGEVFTSMLGLYFNN  120
    OsHKT1;1-V7  (P)   61  GKPGPKDLDLLFTSVSTLTVSSMATVEMEDLSDRQLWVLILLMLMGGEVFTSMLGLYFNN  120

    OsHKT1;1-V7  (N)  121  ANANRNENSQRSLPSISLDIESNSPANNGDHKITECGQSEETMSQNQRFRQCRLLTRIQL  180
    OsHKT1;1-V7  (P)  121  ANANRNENSQRSLPSISLDIESNSPANNGDHKITECGQSEETMSQNQRFRQCRLLTRIQL  180

    OsHKT1;1-V7  (N)  181  PEVVEA                                                        186
    OsHKT1;1-V7  (P)  181  PEVVEA                                                        186

I   OsHKT1;1-V8  (N)    1  MHPPSLVLDTLKRIKLYIAMKLLLPNSEVPRIYWEKAQHLCGFLSMKLISRARCVNLKPQ  60
    OsHKT1;1-V8  (P)    1  MHPPSLVLDTLKRIKLYIAMKLLLPNSEVLRIYWEKAQHLCGFLSMKLISRARCVNLKPQ  60

    OsHKT1;1-V8  (N)   61  GKPGPKDLDLLFTSVSTLTVSSMATVEMEDLSDRQLWVLILLMLMGGEVFTSMLGLYFNN  120
    OsHKT1;1-V8  (P)   61  GKPGPKDLDLLFTSVSTLTVSSMATVEMEDLSDRQLWVLILLMLMGGEVFTSMLGLYFNN  120

    OsHKT1;1-V8  (N)  121  ANANRNENSQRSLPSISLDIEFNSPANNGDRKITECGQSEETMSQNQKGSQFLLIH      176
    OsHKT1;1-V8  (P)  121  ANANRNENSQRSLPSISLDIESNSPANNGDHKITECGQSEETMSQNQKGSQFLLIH      176
```

Supplementary Figure S5. Comparisons of amino acid sequences of OsHKT1;1-FL and OsHKT1;1 variants between Nipponbare and Pokkali: OsHKT1;1-FL (**A**), OsHKT1;1-V1 (**B**), OsHKT1;1-V2 (**C**), OsHKT1;1-V3 (**D**), OsHKT1;1-V4 (**E**), OsHKT1;1-V5 (**F**), OsHKT1;1-V6 (**G**), OsHKT1;1-V7 (**H**), and OsHKT1;1-V8 (**I**). GENETYX ver. 16 was used.

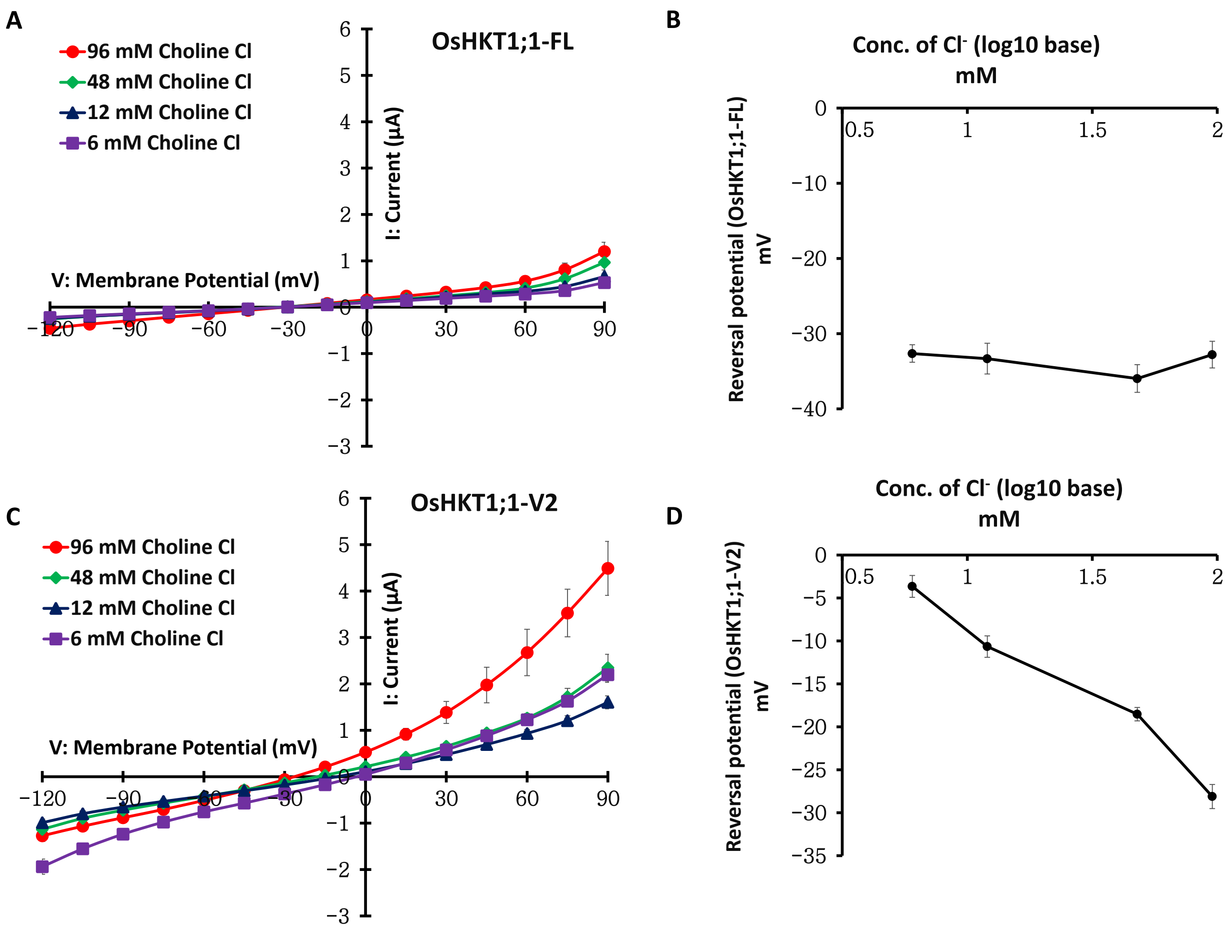

Supplementary Figure S6. Chloride concentration dependency of OsHKT1;1-FL and OsHKT1;1-V2, derived from Nipponbare, and expressed in *X. laevis* oocytes. (**A**, **C**) Current-voltage relationships from oocytes expressing OsHKT1;1-FL and OsHKT1;1-V2, respectively. (**B**, **D**) Log10 base reversal potential analysis of OsHKT1;1-FL and OsHKT1;1-V2, respectively. A series of choline chloride solutions, the concentration of which were from 96 mM to 6 mM, was used as an external bath solution. The basic components of the solutions were 1.8 mM $CaCl_2$, 1.8 mM $MgCl_2$, 1.8 mM mannitol, and 10 mM HEPES, and the pH was adjusted to 7.5 with Tris. 48-, 12-, and 6-mM choline chloride solutions contained 38-, 63-, or 68-mM Mg gluconate, respectively. Oocytes were isolated and injected with 2.5 ng cRNAs/50 nL and then incubated for about 18 h for OsHKT1;1-FL or 4 h for OsHKT1;1-V2 at 18 °C. To obtain current-voltage relationships, voltage steps (2 s) were applied from +90 to −120 mV in 15 mV decrements. Data are presented as means ± SE, *n* = 8-12.

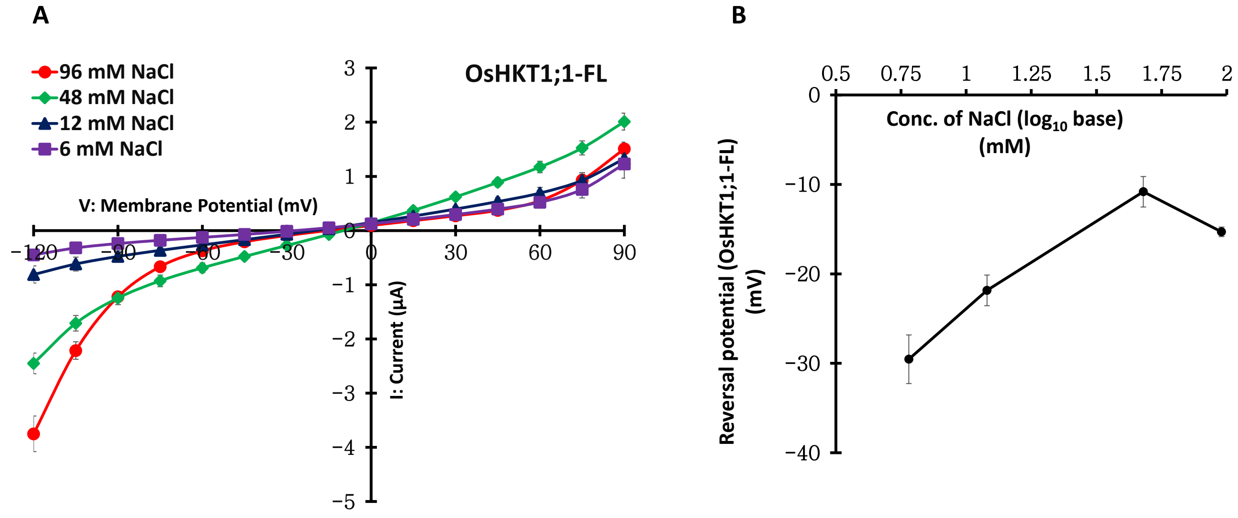

Supplementary Figure S7. Sodium concentration dependency of OsHKT1;1-FL, derived from Nipponbare, expressed in *X. laevis* oocytes. (**A**) Current-voltage relationships of oocytes expressing OsHKT1;1-FL. (**B**) Log10 base reversal potential analysis of oocytes expressing OsHKT1;1-FL. A series solution of NaCl from 96 mM to 6 mM was used as an external solution, the background elements of which were 1.8 mM $CaCl_2$, 1.8 mM $MgCl_2$, 1.8 mM mannitol, and 10 mM HEPES (pH 7.5 with Tris). The 48 mM to 6 mM NaCl solutions contained 38-, 63-, or 68-mM gluconic acid of magnesium (II). Oocytes were isolated and injected with 2.5 ng cRNAs/50 nL and then incubated for approximately 18 h at 18 °C. To get current-voltage relationships, voltage steps (2 sec) were applied from +90 to −120 mV in 15 mV decrements. Data are presented as means ± SE, $n = 10$.
